# High-quality reference transcriptome construction improves RNA-seq quantification in *Oryza sativa* indica

**DOI:** 10.3389/fgene.2022.995072

**Published:** 2022-09-29

**Authors:** Nagesh Srikakulam, Ganapathi Sridevi, Gopal Pandi

**Affiliations:** ^1^ Laboratory of RNA Biology and Epigenomics, Department of Plant Biotechnology, School of Biotechnology, Madurai Kamaraj University, Madurai, India; ^2^ Department of Plant Biotechnology, School of Biotechnology, Madurai Kamaraj University, Madurai, India

**Keywords:** alternative splicing, rice plant, reference transcriptome data, RNA sequencing, Rhizoctonia solani

## Abstract

The Reference Transcriptomic Dataset (RTD) is an accurate and comprehensive collection of transcripts originating from a given organism. It holds the key to precise transcript quantification and downstream analysis of differential expressions and regulations. Currently, transcriptome annotations for most crop plants are far from complete. For example, *Oryza sativa* indica (*O. sativa* indica) is reported to have 40,759 transcripts in the *Ensembl* database without alternative transcript isoforms and alternative splicing (AS) events. To generate a high-quality RTD, we conducted RNA sequencing of rice leaf samples collected at various time points during *Rhizoctonia solani* infection. The obtained reads were analyzed by adopting the recently developed computational analysis pipeline to assemble the RTD with increased transcript and AS diversity for *O. sativa* indica (IndicaRTD). After stringent quality filtering, the newly constructed transcriptome annotation was comprised of 122,968 non-redundant transcripts from 53,695 genes. This study identified many novel transcripts compared to *Ensembl* deposited data that are important for regulating molecular and physiological processes in the plant system. Currently, the assembled IndicaRTD must allow fast quantification of transcript and gene expression with high precision.

## 1 Introduction

Rice is an essential food crop with more than 90% of the world’s rice grown and consumed in the Asia-Pacific region (Papademetriou et al., 2000). Moreover, global rice demand is anticipated to rise by more than 700 million tons by 2025 (Papademetriou et al., 2000). Because of the rise in the world’s population, there is a great demand for a stable food supply. Other challenging factors include pressure on the rice farmlands from urbanization, climate change, and competition from other high-value agriculture that does not enhance rice productivity. Therefore, this global demand needs to be met by increasing rice production in proportion to the growth of the population.

Recently, alternative splicing (AS) emerged as one of the key regulatory mechanisms in the eukaryotic system (Wang S. et al., 2022; Wright et al., 2022). AS plays a significant role in the development and response to biotic and abiotic stressors in plants and therefore provides key revenue to exploit and increase rice production (Calixto et al., 2018; Filichkin et al., 2018; James et al., 2018; Laloum et al., 2018; Zhang G. et al., 2019; Dantas et al., 2019; Qin et al., 2020).

AS allows the production of multiple transcripts from a single genomic locus, which increases transcriptome and proteome diversity (Syed et al., 2012). Events such as exon skipping, intron-retention (IR), alternative donor sites or acceptor sites or their combinations impact the final transcript structure (Reddy et al., 2013; Zhang et al., 2017a). Additionally, an alternative transcription start site (TSS) or polyadenylation (poly-A) site (PAS) may result in either an additional transcript or premature termination generating transcript variants (Sherstnev et al., 2012; Morton et al., 2014). Thus, all variant transcripts may not be functional mRNAs. Even though all of the transcript variants may not encode a functional protein, there should be differential expression at the cellular level. Varied expression of AS transcript variants may influence molecular events by acting as miRNAs sponges, through protein sequestration, or by producing anti-sense RNAs. Another possibility is that AS variants may be subjected to non-sense-mediated mRNA decay (Kalyna et al., 2012; Schweingruber et al., 2013; Hug et al., 2016; Rigo et al., 2019; Raxwal et al., 2020). Considering the possible role in the molecular event, it has been shown that AS impacts various developments and responses to biotic stresses. Recent studies show a massive and rapid AS change that governs the physiological and survival response of plants in response to low temperatures (James et al., 2012; Calixto et al., 2018; James et al., 2018). AS regulations are likely also involved in responses to biotic stresses (Zhang H. et al., 2019; Qin et al., 2020). In the *O. sativa* indica variety, it was found that the OsGBF1 splice variant is upregulated upon salinity stress (Ashwini et al., 2018). AS transcripts of tissue-specific Ser/Arg-rich (SR) genes show varied expression levels in different hormones and stress treatments (Zhang et al., 2013). OsNPF6.5 nitrate-transporter gene splice variant NRT1.1B is also associated with a higher nitrate uptake mechanism (Hu et al., 2015), and OsFe-SOD isoforms are upregulated in both vegetative and reproductive tissues by light induction (Feng et al., 2006).

Advancements in the sequencing technology and tool development have helped construct high-quality, more diverse, and high-confidence transcript references in *Arabidopsis thaliana*, AtRTD, and AtRTD2 (Zhang et al., 2015; Brown et al., 2017; Zhang et al., 2017b). The pipeline for the construction of AtRTD2 includes stringent filtering and quality control measures not only based on plant intron and splicing characteristics to reduce the number of transcripts with false splice junctions (SJs), but also addressing issues such as redundancy, fragmentation, and misannotations at the 5′ and 3′ end. A similar approach was followed for barley for high-quality reference transcriptome data, which achieved improved quantification accuracy through experimental validations (Rapazote-Flores et al., 2019).

The existing and available public transcriptome annotations for two major rice varieties, *Oryza sativa* ssp. japonica and *O. sativa* indica, have been deposited with 45,722 and 42,031 transcripts, respectively. The Transcriptome ENcyclopedia Of Rice database (TENOR-db) is part of the rice annotation project (rap-db), which is an actively updated source for the japonica variety deposited with 23,943 full-length protein-coding cDNAs and 9336 partial protein-coding cDNAs (Ohyanagi et al., 2006; Kawahara et al., 2016). A total of 3.5 billion single-end sequencing raw reads with a 76 bp read length were used to construct the TENOR-db. TOPHAT2 sequence aligner (Ghosh and Chan, 2016) and Cufflinks reference transcriptome assembly tools were used for assembling the reads. The *Ensembl* deposited transcriptome annotation for the indica variety was created with publicly available sequence tagged sites (STSs), full-length cDNAs, and expressed sequence tags (ESTs) (Yu et al., 2002; Cunningham et al., 2019). However, studies have reported AS transcripts in the indica and japonica varieties, which do not mention the high-quality transcriptome data (Lu et al., 2010; Zhang et al., 2010; Zhang G. et al., 2019; Schaarschmidt et al., 2020; Wang X. et al., 2022; Hasan et al., 2022; He et al., 2022). The approximate size of the rice diploid genome is 500 MB compared to the 135 MB of *A. thaliana*, which recently reported 82,190 non-redundant transcripts from 34,212 genes in the AtRTD2 database (db) (Brown et al., 2017; Zhang et al., 2015, 2017). These statistics show that many splicing events could be missing in rice transcriptome datasets. By employing the novel pipeline used for AtRTD2 construction based on the reference genome and taking advantage of paired-end sequencing of greater length and depth, we constructed IndicaRTD and generated 122,968 non-redundant transcripts from 53,695 genes, which represents a significant improvement to the current *Ensembl* annotation.

## 2 Methods

### 2.1 Plant material collection, RNA extraction, and sequencing

RNA-seq data (paired-end 2 × 100 bp) were generated for leaf tissue of *O. sativa* indica infected with *R. solani* (BioProject ID: PRJNA725331). All plants were grown in the greenhouse at 32 °C for 40 days before infection. Leaf samples were collected at 12 h intervals up to 72 h post-infection (hpi) along with control (mock-inoculated) samples. An RNeasy Plant Mini Kit was used for RNA isolation (Qiagen, Hilden, Germany). RNA quality and quantification were checked using a Nanodrop ND-1000 (Thermo Scientific, Waltham, MA, United States), Qubit fluorometer (Thermo Scientific, Waltham, MA, United States), and Bioanalyzer 2100 (Agilent, Santa Clara, CA, United States). RNA samples were confirmed to have an RNA integrity number (RIN) above 7 to proceed with library preparation using the NEBNext mRNA library preparation kit (New England Biolabs, Ipswich, MA, United States) according to the manufacturer’s protocol and then followed by RNA sequencing. A total of 18 libraries of RNA-seq runs were obtained on the Illumina HiSeq 2500 platform (Illumina, San Diego, CA, United States).

### 2.2 Quality filtration and genome mapping

tRNA-seq raw data quality filtration was performed to remove low-quality reads and adapter sequences followed by genome mapping to identify the known and novel SJs. RNA-seq reads of 18 libraries were quality filtered with AdapterRemoval (v2.3.0) [--minquality 25 --adapter-listAdapter.txt] (Schubert et al., 2016). The genome for the indica variety was downloaded from the *EnsemblPlant* database (ftp://ftp.ensemblgenomes.org/pub/plants/release-44/fasta/oryza_indica/dna/). Genome index files were created using the STAR alignment tool (v2.7.2b), and quality filtered reads were mapped to the genome index with a 2-pass mapping strategy (Dobin et al., 2013; Dobin and Gingeras, 2016; Zhang et al., 2017a). The first-pass mapping mode was performed with the parameters --sjdbOverhang 100 --outSAMprimaryFlag AllBestScore --outFilterMismatchNmax 2 --outSJfilterCountTotalMin 10 5 5 5 --outSAMstrandField intronMotif --outFilterIntronMotifs RemoveNoncanonical --alignIntronMin 60 --alignIntronMax 6000 --outFilterScoreMinOverLread 0 --outFilterMatchNminOverLread 0 --alignMatesGapMax 400. The novel SJs of the first-pass mapping were used for generating the genome index files for second-pass mapping with zero mismatches [--outFilterMismatchNmax 0] and allowed accurate mapping around the splice junctions and the rest of the parameters were similar to the first-pass mapping (Supplementary Flow Chart S1).

The output file of STAR aligner (sj.out.tab) consists of detailed information about SJ including coordinates, overhang, the number of uniquely mapping reads crossing the junction (column 7), and number of multi-mapping reads crossing the junction (column 8). The SJ files of each library from the second pass mapping were used to infer high-confidence SJs that are well supported by the mapped reads. To retrieve the high-confidence SJs, we considered the following features: 1) SJs with canonical intron motif, 2) at least one uniquely mapping read count crossing the SJ, and 3) 0 mismatch read alignment.

### 2.3 Assembly and merging

Three different reference-based transcriptome assemblers, Cufflinks (v2.2.1) (Trapnell et al., 2010), StringTie2 (v2.0.1) (Kovaka et al., 2019) and Scallop (v0.10.3) (Shao and Kingsford, 2017) were used to assemble the reads with the default parameters. The sorted BAM files generated from STAR alignment were used to assemble the reads of each library. The resulting transcripts (GTF files) for 18 libraries of each assembler tool were merged with three different merging tools, Cuffmerge (v2.2.1) [--min-isoform-fraction 0] (Ghosh and Chan, 2016), StringTie2-Merge (v2.0.1) (StringtieM) [-F 0 -T 0 -f 0 -g 0] (Kovaka et al*.,* 2019), and Taco (v0.7.3) [--gtf-expr-attr RPKM --filter-min-expr 0 --isoform-frac 0 --max-isoforms 0] (Niknafs et al., 2017).

### 2.4 Evaluation of transcriptome annotation

We evaluated the assembled and merged transcriptome annotation files to identify the best performance assembler and merging tool. Exon and intron coordinates were extracted using the transcriptome annotation GTF files generated by three different assemblers along with three various merging tools as mentioned in Section 2.3. Intron coordinates were constructed using the construct_introns function from gread R library (https://rdrr.io/github/asrinivasan-oa/gread/) (v0.99.3).

To compare transcripts produced by different combination tools, we generated Venn diagrams of overlapping non-redundant transcripts by exon and intron coordinates of each assembler with three merging tools. We also analyzed merging tools annotation files with transcript quantification tools such as Salmon (v1.3.0) (Patro et al*.,* 2017) [-i index folder -l ISR -1 fastq1 -2 fastq2 --gcBias --seqBias --posBias --dumpEqWeights -o output] and Kallisto (v0.46.2) (Bray et al., 2016) [-i Kallisto_index --bias fastq file -o output file]. All 9 annotation files from 3 merging tools (Cuffmerge, StringtieM, and Taco) for 3 assemblers (Cufflinks, StringTie2, and Scallop) were used for the transcript quantification evaluation.

### 2.5 Filtration and validation of transcriptome annotation

Based on the evaluation analysis, StringtieM was used to merge the raw assembly transcript annotations of various assemblers. We filtered the transcripts with non-canonical and poorly supported SJs (low abundance and short overhang length of spliced alignment) to improve the annotation quality. We used in-house build scripts to create the SJ database (SJdb) from STAR second-pass mapping output (sj.out) files. The resulting annotation was merged using StringtieM with the *Ensembl* transcriptome dataset to create IndicaRTD.

We adapted the junction coverage compatibility (JCC) analysis (Soneson et al., 2019) for further validation of the accuracy of the IndicaRTD. Initially, we created the BSgenome library for the *O. sativa* indica genome downloaded from the *EnsemblPlant* database using the BSgenomeforge R function (Pagès, 2019). To calculate JCC scores, we first fit a fragment-level bias model using the fitAlpineBiasModel function of the JCC R package and a wrapper for alpine Bioconductor package functions for each library separately (Love et al., 2016). We used a set of single-isoform genes with a length between 600 bp and 7,000 bp and between 500 and 10,000 assigned reads to fit the bias model. The fragment bias model fits into the model to predict the coverage profiles for each transcript in the reference catalog using the predictTxCoverage function of the JCC R package. Later, the scaleTxCoverages function of the JCC R package was used to measure the coverage profiles by the transcript abundance estimates extracted by the Salmon alignment-based method to determine the predicted number of reads covering each position in the transcript. Also, this step extracts the sum of the predicted number of reads for each unique junction across all transcripts. We extracted the number of reads observed for each junction from the STAR aligner output (SJ.out.tab) file for each library. We also used the combineCoverages function from the JCC R package combined with both the predicted junction coverages from the scaleTxCoverages function and observed junction coverages from STAR alignment. This also provides information on transcript abundances at the gene level and includes information about the number/fraction of uniquely and multi-mapping reads passaging each junction. Further calculateJCCScores function was used to estimate the JCC scores of each gene for each library. A similar method was followed for *Ensembl* RTD to calculate the JCC scores. Density plots were generated for the JCC scores of both IndicaRTD and *Ensembl* RTD.

We also performed the validation of IndicaRTD compared with *Ensembl* RTD with two transcript quantification tools such as Salmon (v1.3.0) (Patro et al*.,* 2017) [-i index folder -l ISR -1 fastq1 -2 fastq2 --gcBias --seqBias --posBias --dumpEqWeights -o output] and Kallisto (v0.46.2) (Bray et al., 2016) [-i kallisto_index --bias fastq file -o output file]. Both Salmon and Kallisto tools were developed for the fast and accurate transcript quantification compared to other currently available quantification tools such as cufflinks and TopHat. (Zhang et al., 2017b; Sarantopoulou et al., 2021) (https://learn.gencore.bio.nyu.edu/rna-seq-analysis/salmon-kallisto-rapid-transcript-quantification-for-rna-seq-data/). Furthermore, we listed the aligned read and its transcript percentages for each of the 18 libraries for the transcriptome annotation for both the Salmon and Kallisto tools.

## 3 Results

### 3.1 Quality filtration and genome mapping

After quality filtration was performed with a minimum Phred score of 25 and removal of adapter contamination from raw reads, ∼100% reads were retained in each library with 98 nt–99 nt average read length at each end (Supplementary Table S1). Approximately 88–94% of filtered reads were shown to be uniquely mapped to the indica genome (Supplementary Tables S2, S3). STAR first-pass mapping generated a total of 317,401 SJs. Among them, 213,128 are shown as unannotated novel canonical SJs with at least one uniquely mapped read count along with 101,766 annotated canonical SJs with at least one uniquely mapped read count from *Ensembl,* and the rest of the 2,507 SJs have multi-mapped reads. During STAR second-pass mapping, a total of 306,789 SJs were generated. Among them, 1,862 were shown as unannotated novel canonical SJs with at least one uniquely mapped read count, 300,875 were shown as annotated canonical SJs with at least one uniquely mapped read count from *Ensembl,* and first-pass mapping SJs and the rest of the 4,052 SJs had multi-mapped reads (Table 1). Filtering based on the canonical SJ intron sequence motifs with at least one uniquely mapped read count crossing the SJ and 0 mismatch read alignment generates 302,737 unique SJs. The number of SJs for each canonical splice site was extracted (Supplementary Table S4). GT/AG and its equivalent CT/AC splice signals are found in 296,962 (∼98%) SJs compared to other canonical splice signals GC/AG and CT/GC (5,473), AT/AC, and GT/AT (302). A total of 82.77% of total SJs are found in at least two libraries (Supplementary Table S5).

**TABLE 1:** STAR first and second-pass mapping splice junction (SJ) statistics. RNA-seq clean reads were mapped on the *Oryza sativa* Indica genome using STAR aligner, which generated the possible SJs. The total number of unique SJs was given with uniquely and multi-mapped reads crossing the SJs. The numbers of both canonical and annotated SJs with at least 1 uniquely mapped read crossing the junction are given.

STAR alignment splice junctions (SJs) statistics		
Feature	First-pass mapping with 2 mismatches	Second-pass mapping with 0 mismatches
Unique no. of novel SJs (Uniquely + multi-mapped reads)	214,063	2,031
Unique no. of annotated SJs (Uniquely + multi-mapped reads)	103,338	304,758
Unique no. of novel canonical SJs with at least 1 uniquely mapped read count	213,128	1,862
Unique no. of annotated canonical SJs with at least 1 uniquely mapped read count	101,766 (*Ensembl*)	300,875 (*Ensembl*+1st pass novel)
Total unique no. of canonical SJs with at least 1 uniquely mapped read count	314,894	302,737
Total no. of unique SJs (Uniquely + multi-mapped reads)	317,401	306,789

### 3.2 Assembly and merging

The number of genes and transcripts were calculated and plotted for each library generated by the three different reference-based transcriptome assemblers such as Cufflinks, StringTie2, and Scallop (Supplementary Figures S1, S2). Cufflinks assembled 47,154 to 50,555 genes and 76,593 to 83,054 transcripts per sample, while StringTie2 and Scallop assembled 25,952 to 36,952 genes and 46,588 to 61,541 transcripts per sample (Supplementary Table S6). Cufflinks assembled 25–45% more genes and 24–39% more transcripts compared to other assemblers. The number of mapped reads by the STAR aligner is shown on the secondary *Y*-axis. The number of mapped reads was shown to be distributed between 23.2 million to 46.9 million. The number of assembled genes and transcripts does not seem significantly impacted by the depth of sequencing at this range.

The detailed observation of the excess number of assembled genes and transcripts of Cufflinks revealed that it generated new transcripts with false splice junctions. The total unique number of SJs of Cufflinks, StringTie2, and Scallop across all 18 libraries are 376,001, 180,118 and 169,757, respectively (Supplementary Table S7). Cufflinks generated 52 and 54% more SJs compared to StringTie2 and Scallop, respectively. Later, we decided to compare the SJs generated by the individual assembler with second-pass mapping of STAR aligner-produced SJs. Overall, Cufflinks, StringTie2, and Scallop match 45, 98, and 98% of SJs with STAR second-pass mapping generated SJs. As anticipated, Cufflinks consisted of ∼55% more false SJs than StringTie2 (2%) and Scallop (2%).

Later, merging all of the assembled data for the 18 libraries for each assembler was performed with three different merging tools including Cuffmerge, StringtieM, and Taco. Cuffmerge generated 54,387, 47,182, and 49,686 genes and 170,241, 116,172, and 118,487 transcripts for Cufflinks, Scallop, and StringTie2 assemblers, respectively. However, Taco produced 48,857, 35,167, and 43,929 genes and 126,236, 132,100, and 70,413 transcripts for Cufflinks, Scallop, and StringTie2 assemblers, respectively. Similarly, StringtieM generated 51,012, 34,455, and 43,564 genes and 256,275, 119,581, and 101,407 transcripts for Cufflinks, Scallop, and StringTie2 assemblers, respectively (Supplementary Table S8). For Cufflinks assembled data, the percentage of transcripts produced by StringtieM was 33 and 50% higher than Cuffmerge and Taco, respectively, though the percentage of genes was 6% less than Cuffmerge and 4% higher than Taco. The Scallop assembled data shows 14 and 2% higher transcripts and 36 and 2% lesser genes than Cuffmerge and Taco, respectively. Similarly, for StringTie2 assembled data, StringtieM shows 16% fewer transcripts than Cuffmerge. Still, it offers a 30% higher number of transcripts than Taco merged data and 14 and 0.8% fewer genes than Cuffmerge and Taco, respectively. Among the three merge tools, StringtieM shows a more significant number of AS variants from a lower number of genes compared with the other two merge tools, Cuffmerge and Taco.

The mono-exonic transcripts are mRNAs with a single stretch of a protein-coding region and without any non-coding regions to splice out. The number of mono-exonic transcripts for the merged annotation of Cufflinks assembled data shows 25,516, 23,735, and 21,350 for Cuffmerge, Taco, and StringtieM, respectively (Supplementary Table S9). Similarly, for the Scallop and StringTie2 assembled data, the number of mono-exonic transcripts were distributed from 8,865 to 18,011 for the different merged annotations. Though Cufflinks data shows a greater number of mono-exonic transcripts, Taco merged annotation of the StringTie2 assembler annotation files show a high percentage of mono-exonic transcripts compared to the other merge tools.

### 3.3 Evaluation of transcriptome annotation

**FIGURE 1:**
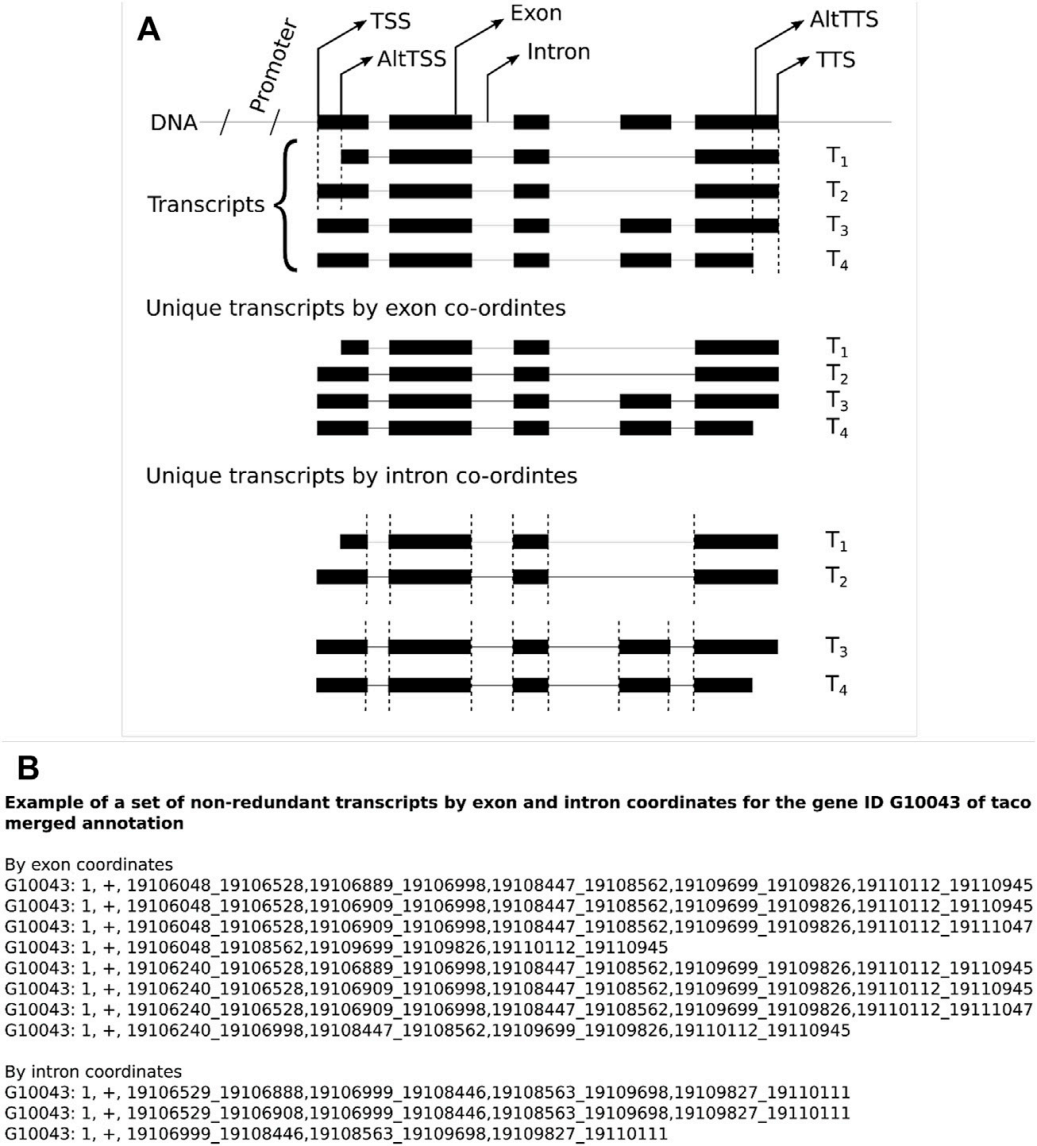
**(A)** Illustration of the usage of exon and intron coordinates for non-redundant transcripts to evaluate transcript GTF files. **(B)** An example set of non-redundant transcripts for the gene ID G10043 for the Taco merged annotation file by exon and intron coordinates.

A comparison of non-redundant transcripts was performed for the three merge tools (Cuffmerge, Taco, and StringtieM) by exon and intron coordinates for each assembler of Cufflinks, Scallop, and StringTie2. When using intron coordinates, the variations at the 5′ and 3′ end of the transcript are not considered; two transcripts sharing the exact intron coordinates are considered the same transcript. While using exon coordinates, two transcripts must be the same from start to end (Figure 1A). The example list shows the non-redundant transcripts by exon and intron coordinates for the gene ID G10043 from the GTF file of Taco merged annotation along with chromosome and strand information (Figure 1B).

The transcript structures can be represented either by the intron or exon coordinates. The difference is that transcripts represented by intron coordinates ignore variations of the TSS and alternative PAS, which standard RNA-seq are not equipped to capture. We compared transcript structures by exon and intron coordinates among the different assemblers and the different merge tools. While considering the exon coordinates of transcripts, both the 5′ and 3′ UTR region coordinates vary among the transcripts of a gene, which permits a more significant number of unique transcripts. These coordinates can be avoided by choosing the intronic region coordinates and collapsing the duplicate transcripts to compare among the merge tools. While using the intron coordinates for the transcripts, the number of overlapping non-redundant transcripts among three assemblers (Cufflinks, Scallop, and StringTie2) for each merging tool Cuffmerge, Taco, and StringtieM show 43,195, 18,191, and 25,460 compared to exon coordinates 42,144, 55, and 147, respectively (Supplementary Table S10; Supplementary Figure S3).

The distribution of overlapping non-redundant transcripts among the three assemblers by intronic coordinates was calculated (Supplementary Figure S4). StringtieM produces the highest number of total non-redundant transcripts, 347,014 among the three merge tools for the three assemblers by intron coordinates. These statistics show that using intron coordinates is the best for transcript comparison.

The total number of introns corresponding to the length distribution was analyzed for each annotation file (Supplementary Table S11). Approximately 90% of introns in nine merged transcriptome annotations occur at ≤ 1000 nt length (Supplementary Figures S5, S6).

The distribution in the number of isoforms per number of genes was plotted for 1) Cuffmerge, 2) StringtieM, and 3) Taco (Supplementary Figure S7). The Cufflinks assembler shows a more significant number of genes with ≥ 2 isoforms/gene compared to the other two assemblers such as StringTie2 and Scallop.

The overlapping number of unique transcripts by intron coordinates were estimated for all 18 libraries for each assembler (Supplementary Figure S8). In addition, the overlapping number of unique transcripts by intron coordinates for the nine annotation files generated by three merging tools for the raw assembly of three assemblers were estimated (Supplementary Figure S9). StringtieM showed better performance with most of the transcripts matching with the parent annotation transcripts compared to other merging tools.

Salmon (v1.3.0) and Kallisto (v0.46.2) read mapping statistics for nine transcript annotation files of three merging tools (Cuffmerge, StringtieM, and Taco) and for three assemblers (Cufflinks, StringTie2, and Scallop) were calculated (Supplementary Table S12). For all annotations, the percentages of the mapping rate is distributed between 86.63 and 93.94% for Salmon and 91.47–95.27% for Kallisto aligners. Similarly, percentages of transcripts aligned by clean reads are distributed between 88.37 and 99.86% for Salmon and 82.77–98.04% for Kallisto aligners. The higher the percentage of transcript alignment with a high percentage of reads, the better the sensitivity and specificity.

To investigate how SJs and transcripts change before and after the merge step, we calculated the precision and recall values for merging transcriptome annotation with the raw annotation transcripts of the assemblers before the merge. Although there is a significant overlap between raw assembly and merged assembly in terms of transcript structures, we observed that some of the raw assembly transcripts disappeared after the merge. Similarly, some merge tools generate new transcripts not in the raw assemblies. Precision represents the closeness of the merging annotation calculated by taking the ratio of the number of non-redundant overlapping transcripts of merging annotation in the raw assembly annotation files and the total number of non-redundant transcripts in merging annotation. The recall represents the recovery rate of the merging annotation calculated using the ratio of the number of non-redundant overlapping transcripts of the merging annotation in the raw assembly annotation files and the total number of non-redundant transcripts in the raw assembly annotation files (Supplementary Figure S10). Scatter plots were generated using the precision and recall values on both the *X* and *Y*-axis, respectively.

A Scatter plot of the precision and recall values of the unique intronic segment coordinates (Supplementary Figure S11A) and unique transcripts by intron coordinates (Supplementary Figure S11B) demonstrates that StringtieM is the best performance merge tool. StringtieM shows transcript recall rates of 58, 77, and 55% for Scallop, Cufflinks, and StringTie2 assemblers, respectively, and compared with other merge tools, Cuffmerge and Taco, which are distributed from 33 to 52%. Also, the precision levels of transcripts of the StringtieM are much higher (above 95%) for the three different assemblers compared to Cuffmerge and Taco, which are distributed from 63 to 92%.

### 3.4 Filtration and validation of transcriptome annotation

Based on precision and recall values, StringtieM annotation shows the best true positive rate and closeness with raw assembly compared with Cuffmerge and Taco. Therefore, the StringtieM merge annotation of the raw assemblies of different assemblers was used for further analysis. We used the in-house built shell script to create the SJdb with features such as canonical SJ, the number of unique mapping reads crossing the junction (≥5 reads), and the maximum spliced alignment overhang length (≥10 nt). SJdb was created with 138,532 unique canonical SJs out of 306,789 SJs generated from STAR second-pass mapping (Supplementary Table S13). A total of 102,950 transcripts consisting of these valid SJs were retained out of annotation of 345,918 transcripts. We created the IndicaRTD with a total of 122,968 transcripts after merging with StringtieM with 102,950 transcripts and the *Ensembl* transcriptome dataset.

JCC was implemented to validate the completeness of the reference transcriptomic data set. JCC scores of the genes measure the agreement of the predicted junction coverage of the transcript abundance estimation from the Salmon method, and the observed number of junction reads from the STAR aligner. A higher JCC score represents a higher disagreement between the predicted junction coverage and the observed number of junction reads. It has been used to identify genes with poor annotations, such as missing transcript isoforms and missed annotations at the 5′ and 3′ UTR regions. Additionally, we use the JCC score to measure the quality of the transcriptome annotations. Genes with a lower JCC score represent the transcript annotation with a higher quality and are expected to have a higher level of agreement between predicted junction coverage and the observed number of junction reads.

**FIGURE 2:**
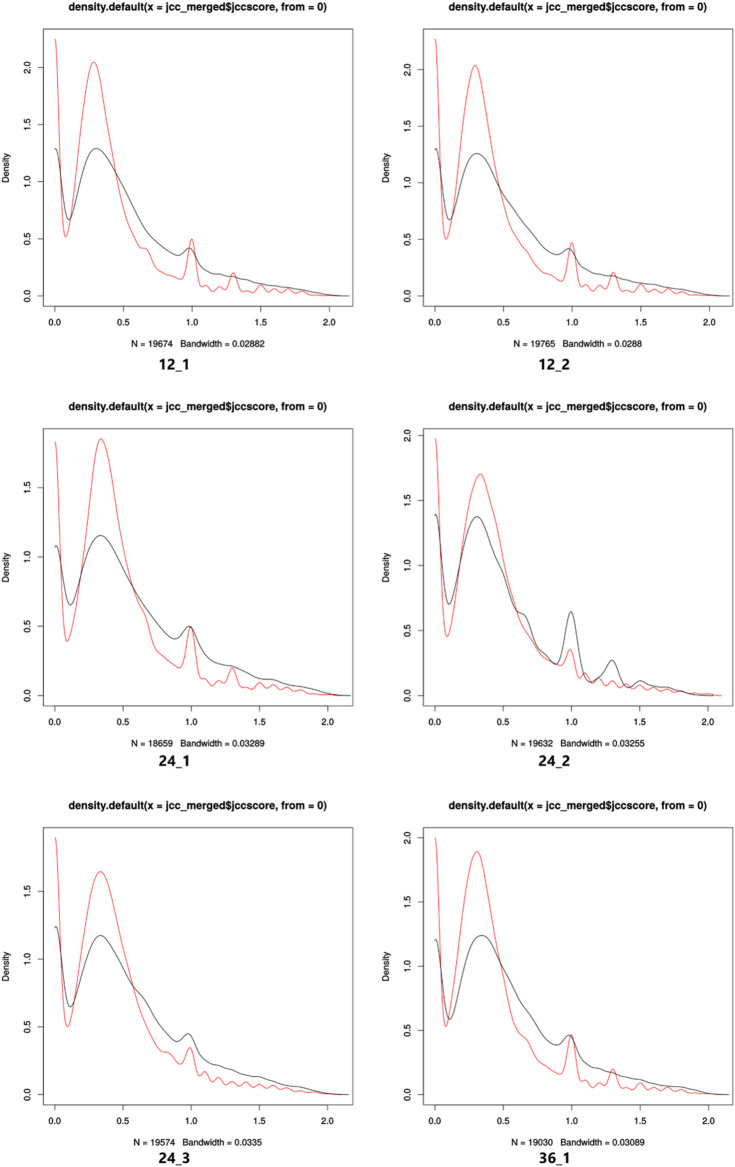
Density plots of the JCC scores of IndicaRTD (red line) and *Ensembl* RTD (black line) genes for each sequencing library. A comparison of the distribution of genes with JCC scores for both IndicaRTD and *Ensembl* RTD annotation for 12_1, 12_2, 24_1, 24_2, 24_3, and 36_1 sequencing libraries are shown. The *X*- and *Y*-axis represent the JCC score and gene density, respectively.

In this study, the IndicaRTD shows more genes with a better JCC score (≤0.6) compared to the *Ensembl* RTD in all sequencing libraries (Figure 2; Supplementary Figure S12). Therefore, the IndicaRTD from the current study can provide a more accurate quantification of transcript and gene expressions using RNAseq data. Thus, the IndicaRTD is currently available as a more comprehensive and accurate transcript annotation.

We assessed the percentage of clean reads aligned to the Indica and *Ensembl* RTD annotations and the number of transcripts mapped by reads for both the Salmon and Kallisto quantification tools (Table 2). In this study, IndicaRTD shows ∼90% of reads mapped in all 18 libraries compared to ∼77% of *Ensembl* RTD. Also, the number of transcripts showed expression (with reads support) >2-fold when using IndicaRTD compared to *Ensembl* RTD. These statistics show that the current study, IndicaRTD annotation, is more reliable for expression analysis than the *Ensembl* RTD.

**TABLE 2:** Comparison of IndicaRTD and Ensembl RTD. **(A)** The table shows the percentages of reads aligned with the transcripts and the number of transcripts mapped by the RNA-seq reads by the Salmon mapping tool for both *Ensembl* and IndicaRTD annotation. **(B)** The table shows the percentages of reads aligned to the transcripts and the number of transcripts mapped by the RNA-seq reads by the Kallisto mapping tool for both *Ensembl* and IndicaRTD annotation.

(A) Salmon alignment				
	Read mapping rate for the transcripts		Number of transcripts with read mapping	
	*Ensembl* RTD	Indica RTD (current study)	*Ensembl* RTD	Indica RTD (current study)
12_1	74.31	90.60	27224	78192
12_2	73.96	90.66	27363	78661
24_1	69.85	87.33	25909	72772
24_2	77.64	90.94	27206	75207
24_3	75.71	90.75	27240	76214
36_1	79.30	91.73	26185	72401
36_2	72.42	90.12	26104	74237
48_1	76.19	90.93	26127	69162
48_2	69.41	89.77	26530	74537
60_1	77.07	90.99	26835	76159
60_2	75.04	90.53	26534	74710
60_3	75.10	90.68	26801	77213
72_1	73.96	90.44	26741	76542
72_2	75.42	91.08	25781	73774
72_3	73.81	90.57	26941	77593
C1	77.64	90.94	27206	75204
C2	73.41	88.37	26996	77617
C3	72.82	86.20	26893	77717

(B) Kallisto alignment				
	Read mapping rate for the transcripts		Number of transcripts with read mapping	
	*Ensembl* RTD	Indica RTD (current study)	*Ensembl* RTD	Indica RTD (current study)
12_1	75.90	92.38	28833	86141
12_2	75.61	92.47	29023	86698
24_1	71.33	89.48	27591	81954
24_2	79.32	92.83	28836	83944
24_3	77.16	92.60	28752	84531
36_1	80.63	93.43	27706	81598
36_2	74.17	91.73	27869	83194
48_1	77.86	93.02	27796	79450
48_2	70.89	91.75	28249	82805
60_1	78.64	92.93	28418	84911
60_2	76.45	92.40	28114	83040
60_3	76.44	92.31	28400	85364
72_1	75.29	92.10	28443	84861
72_2	76.69	92.65	27344	82277
72_3	75.35	92.34	28703	85746
C1	79.32	92.83	28836	83944
C2	74.57	89.94	28649	85597
C3	77.22	91.81	28635	86757

## 4 Discussion

RTD is the major backbone for accurate gene quantification of RNA-seq data analysis and consists of a list of genes and possible transcript isoforms of an organism (Brown et al., 2017; Zhang et al., 2017a; Rapazote-Flores et al., 2019; Vitoriano et al., 2021). The quality of the transcriptome annotation could help with fast and accurate estimation of transcript expression and AS events using the RNA-seq data with the help of the 3DRNA-seq tool (Chaudhary and Kalkal, 2021; Guo et al., 2021; Vitoriano et al., 2021; Escudero-Martinez et al., 2022). Despite rice being an important crop plant and several attempts being made in the past to improve its transcriptome annotation and AS diversity, IndicaRTD contains a significantly higher number of transcript isoforms (Lu et al., 2010; Zhang et al., 2010; Zhang H. et al., 2019; Schaarschmidt et al., 2020; Wang S. et al., 2022; Hasan et al., 2022; He et al., 2022). Several single gene studies have identified the role of AS transcript isoforms in the eukaryotic system including the plant system specifically in rice (Ganie and Reddy, 2021; Singh and Ahi, 2022; Wright et al., 2022). The new IndicaRTD contains 122,968 non-redundant transcript isoforms from 53,695 genes, and from these, 98,362 (∼80%) AS transcript isoforms were produced from 14,916 (∼48%) multi-exon genes. These high-quality transcripts were generated from a total of 138,532 stringent quality filtered unique canonical SJs including 49,223 (36%) novel SJs. A recent study, which was performed with long-read transcriptome sequencing of *O. sativa* ssp. *Japonica* var Nipponbare, has shown a total of 73,659 SJs along with 12,755 (17%) novel canonical and non-canonical SJs (Hasan et al., 2022). Additionally, we performed a comparison study and showed that IndicaRTD consists of a greater number of genes with a complete number of possible transcript isoforms compared to the currently available *Ensembl* RTD. Here, we demonstrate the significance of the improved IndicaRTD using reference-based alignment quantification tools such as Salmon and Kallisto. The Salmon and Kallisto align approximately 90% of the reads to the IndicaRTD, while the *Ensembl* RTD shows 70–80% read alignment. This scenario shows that many transcript isoforms were missing in the *Ensembl* RTD, which are available in the current IndicaRTD.

A recent study on *A. thaliana* RTD (AtRTD3) construction using single-molecule long-read sequencing technology, such as Pacific Biosciences (PacBio), showed more transcript isoforms with novel SJs compared to AtRTD2 (Zhang et al., 2017b; Zhang et al., 2022). Similarly, a report was published on human transcriptome annotation using long-read sequencing technology that found several novel protein-coding and non-coding transcript isoforms (Kuo et al., 2020). Another report showed an improvement in the barley reference transcriptome (BaRTv2.18) by integrating both short- and long-read sequencing data sets compared to BaRTv1 (Rapazote-Flores et al., 2019; Coulter et al., 2022). A similar approach was adapted to identify the 11,733 and 161,913 transcript isoforms in rice (*O. sativa* L. ssp. *Japonica*) and tomato (*Solanum lycopersicum*), respectively (Zhang G. et al., 2019; Clark et al., 2019). Equivalent and upgraded strategies can also be employed to improve IndicaRTD quality. Other reports have shown that long-read sequencing has been performed for rice plants and several novel AS transcripts were found, but none of them discussed comprehensive transcriptome annotation (Schaarschmidt et al., 2020; Wang X. et al., 2022; Hasan et al., 2022; He et al., 2022). Some drawbacks of the current IndicaRTD are a lack of information to identify TSS, transcription end (polyadenylation) sites (TES), alternative polyadenylation (APA), and the right combination of different TSS, TES, and SJs using the short-read RNA-seq data (Zhang et al., 2017a; Zhang et al., 2022). This leads to miss-assembled transcripts and it can be solved by single-molecule long-read sequencing technologies such as PacBio and Oxford Nanopore sequencing (Zhang H. et al., 2019; Clark et al., 2019; Coulter et al., 2022). However, the high error rate in the long-read sequencing leads to the creation of false SJs (Watson and Warr, 2019; Lima et al., 2020). This can be overcome by short-read sequencing data with an advantage of read depth, which permits the generation of high-confidence SJs (Au et al., 2012; Kovaka et al., 2019; Coulter et al., 2022; Zhang et al., 2022). A recent study identified some flaws while creating RTD with a common reference genome sequence for the different genotype transcriptome sequencing data within a species (Guo et al., 2022). Rice has different genotypes with high-value traits and IndicaRTD can be improved by using the genotype-specific reference genome (Das et al., 2013; Roy et al., 2015; Shrestha et al., 2021).

## 5 Conclusion

In this study, we used a novel transcriptome assembly pipeline to improve the quality of the *Oryza sativa* indica reference transcriptome data. Our analysis showed a significant increase in AS transcripts. Moreover, using the current IndicaRTD, we want to perform differential expression analysis for different biotic and abiotic stress conditions in rice. Our evaluation using Salmon and Kallisto shows better performance of IndicaRTD compared to *Ensembl* RTD. Therefore, the IndicaRTD can be employed for better RNA-seq quantification analysis. The current IndicaRTD can be used as preliminary data and improved by employing long-read sequencing technologies such as PacBio and Nanopore. The generalized process of the RTD assembly pipeline should also be adapted for other eukaryotic organisms to generate species-specific transcriptome annotation.

## Data Availability

The original contributions presented in the study are publicly available in NCBI. This data can be found here: https://dataview.ncbi.nlm.nih.gov/object/PRJNA725331?reviewer=d5stjdq51o0naocnqt43siflg0. Further inquiries can be directed to Dr. Sridevi (ganapathisridevi@yahoo.com).
