# Supplementary material for: High-quality reference transcriptome construction improves RNA-seq quantification in Oryza sativa indica
Source: Front Genet. 2022 Sep 29;13:995072. doi: 10.3389/fgene.2022.995072 (PMC9558114; doi:10.3389/fgene.2022.995072)
Supplement: Supplementary file 1 [file Table1.DOCX]

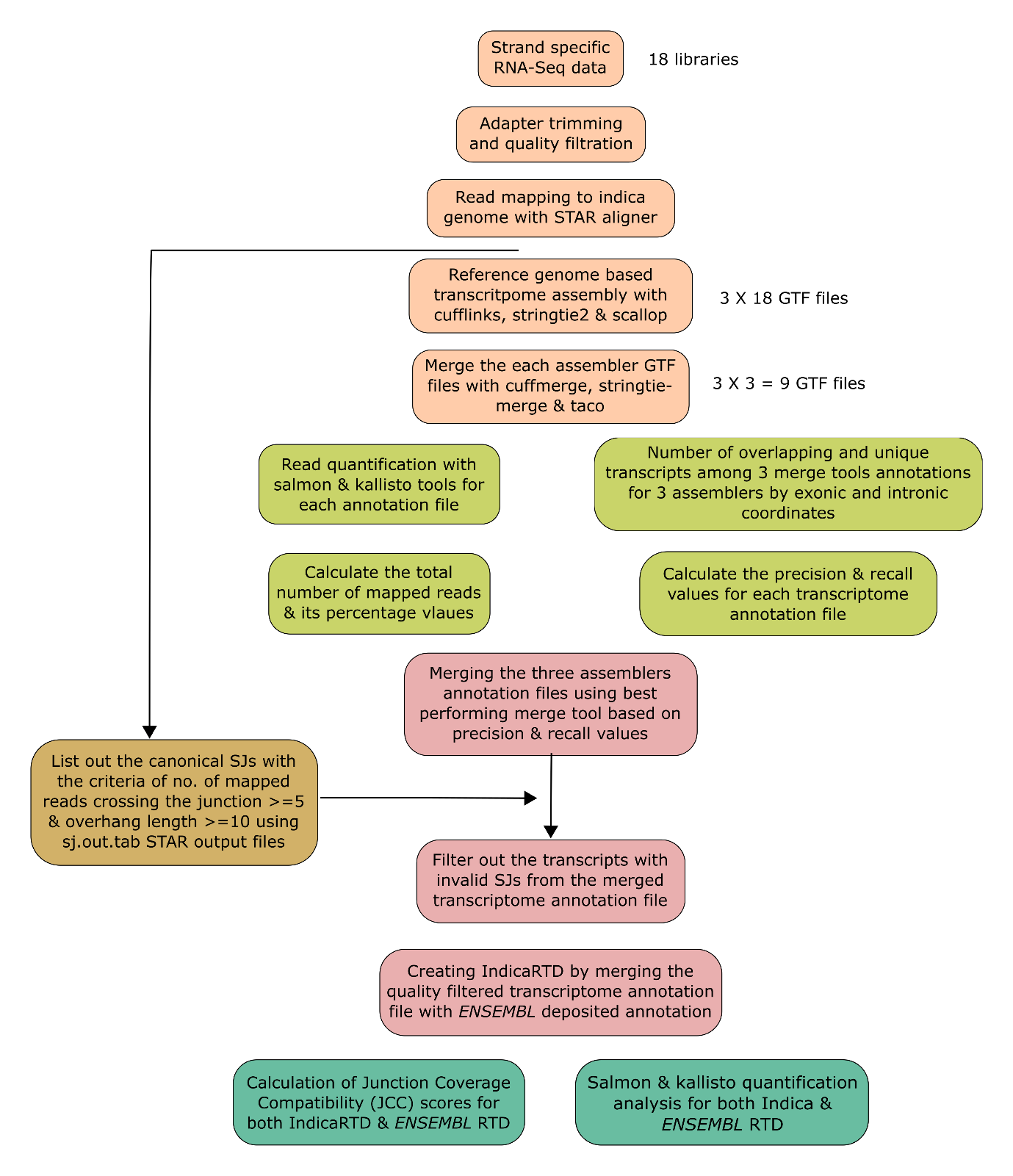


***Flow chart 1****: Schematic representation of bioinformatic analysis pipeline for the IndicaRTD construction and validation.*

***Figure 1****: Number of genes generated by Cufflinks, Scallop and StringTie2 assemblers. The bar chart showing the number of genes produced by the three different assemblers for the 18 libraries. The primary y-axis represents the number of genes, secondary y-axis represents the number of genome aligned reads used for the transcriptome assembly and the x-axis represents the name of the sequencing library.*

***Figure 2****: Number of transcripts generated by Cufflinks, Scallop and StringTie2 assemblers. The bar chart showing the number of transcripts produced by the three different assemblers for the 18 libraries. The primary y-axis represents the number of transcripts, secondary y-axis represents the number of genome aligned reads used for the transcriptome assembly and the x-axis represents the name of the sequencing library.*


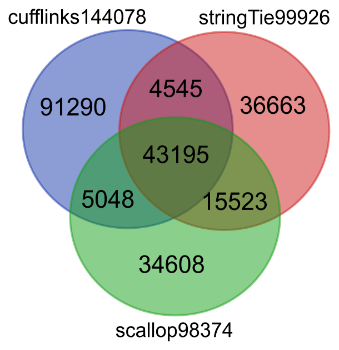

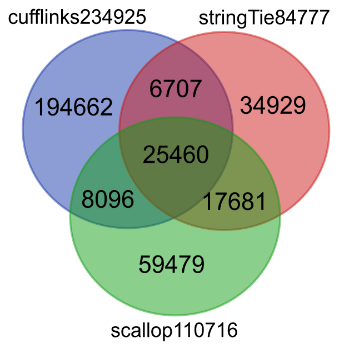

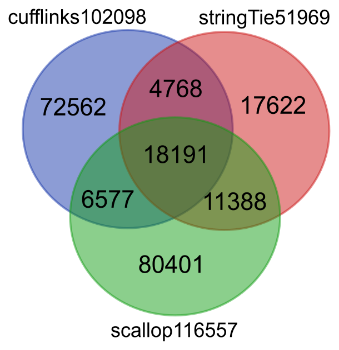

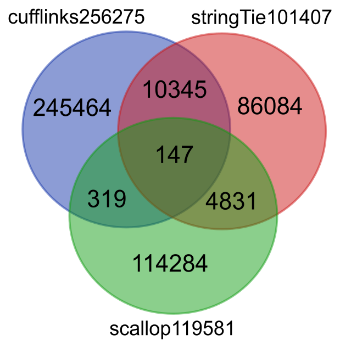

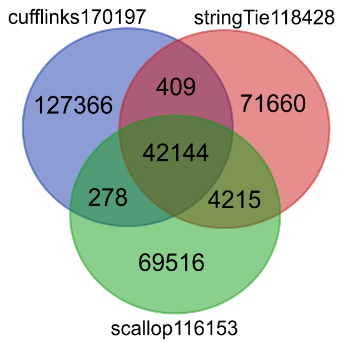

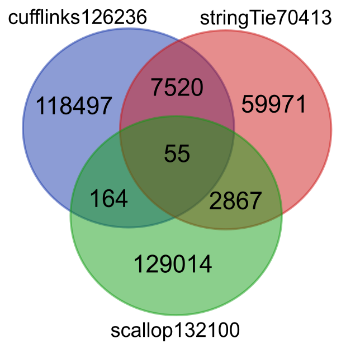


**cuffmerge**

**taco**

**stringtieM**

**By intron**

**By exon**

***Figure 3****: Overlapping unique transcripts by intron and exon coordinates. Venn diagram showing the overlapping unique transcripts by intron and exon coordinates of each merged annotation for three assemblers.*

**

***Figure 4****: Distribution of non-redundant transcripts by intron coordinates. Graph showing the distribution of non-redundant transcripts for each merge tool annotation among three assemblers (cufflinks, scallop and stringtie2) annotation. “1”: transcripts found in any one of the assemblers; “2”: transcripts found in any two of the assemblers; “3”: transcripts found in among three assemblers. X and Y-axis represents the merge tool and number of non-redundant transcripts.*


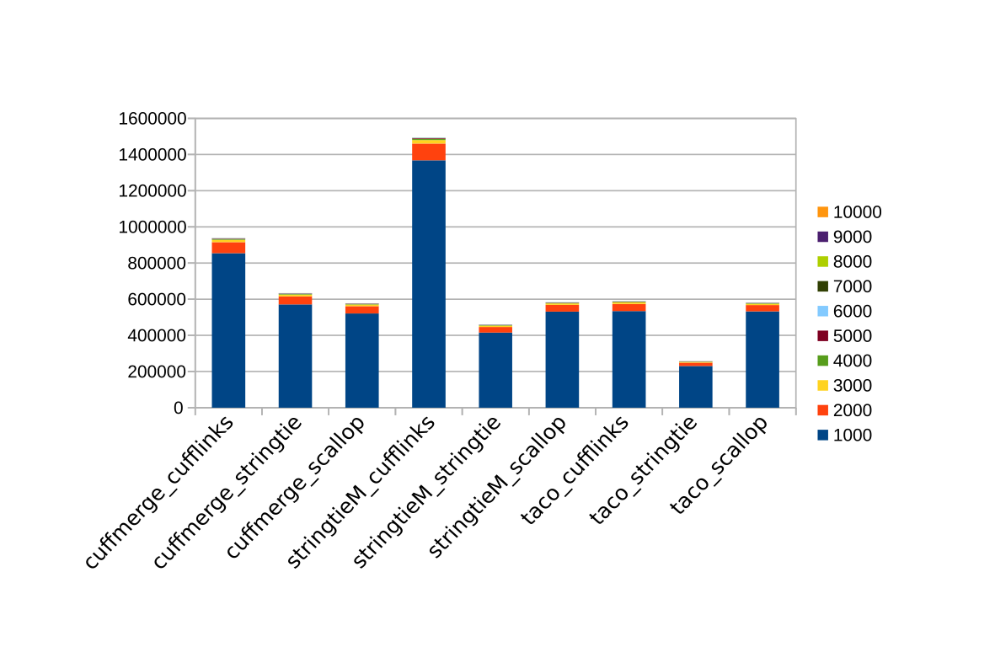


**
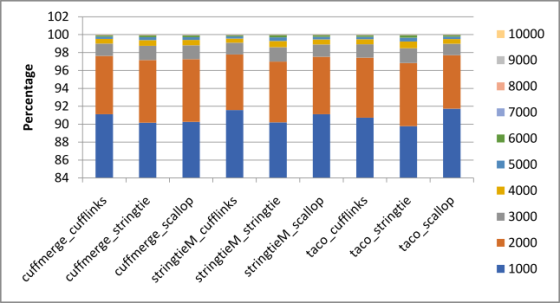
**

***Figure 5****: Length of introns in merged annotation transcripts. The bar chart showing the distribution of the lengths of the introns of the transcripts from merged annotation for three merge tools of each assembly transcriptome annotation. X- and Y-axis represents the merging_assembly tools and number of introns with specific length respectively.*

***Figure 6****: Percentages of lengths of introns in merged assembly. Bar chart showing the distribution of the percentages of the intron lengths of the transcripts from merged annotation for three merge tools of each assembly transcriptome annotation. X- and Y-axis represents the tools used for assembly and merging and percentages of introns with specific length respectively.*

(**a**)

(**b**)

(**c**)

***Figure 7****: Distribution of isoform number per number of genes in merged annotation (a) cuffmerge (b) stringtieM (c) taco. The bar graph showing the number of genes consist of number of isoforms for each merges transcriptome annotation of three different assemblies. X- and Y- axis represents the number of isoforms and number of genes respectively.*


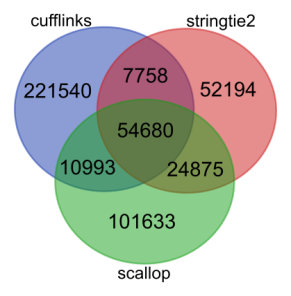


***Figure 8****: Venn diagram of overlapping of unique transcripts by intron coordinates of raw assembly for three assemblers. Venn diagram showing the number of non-redundant transcripts by intron coordinates overlapping among three different assemblies transcriptome annotation of 18 libraries.*


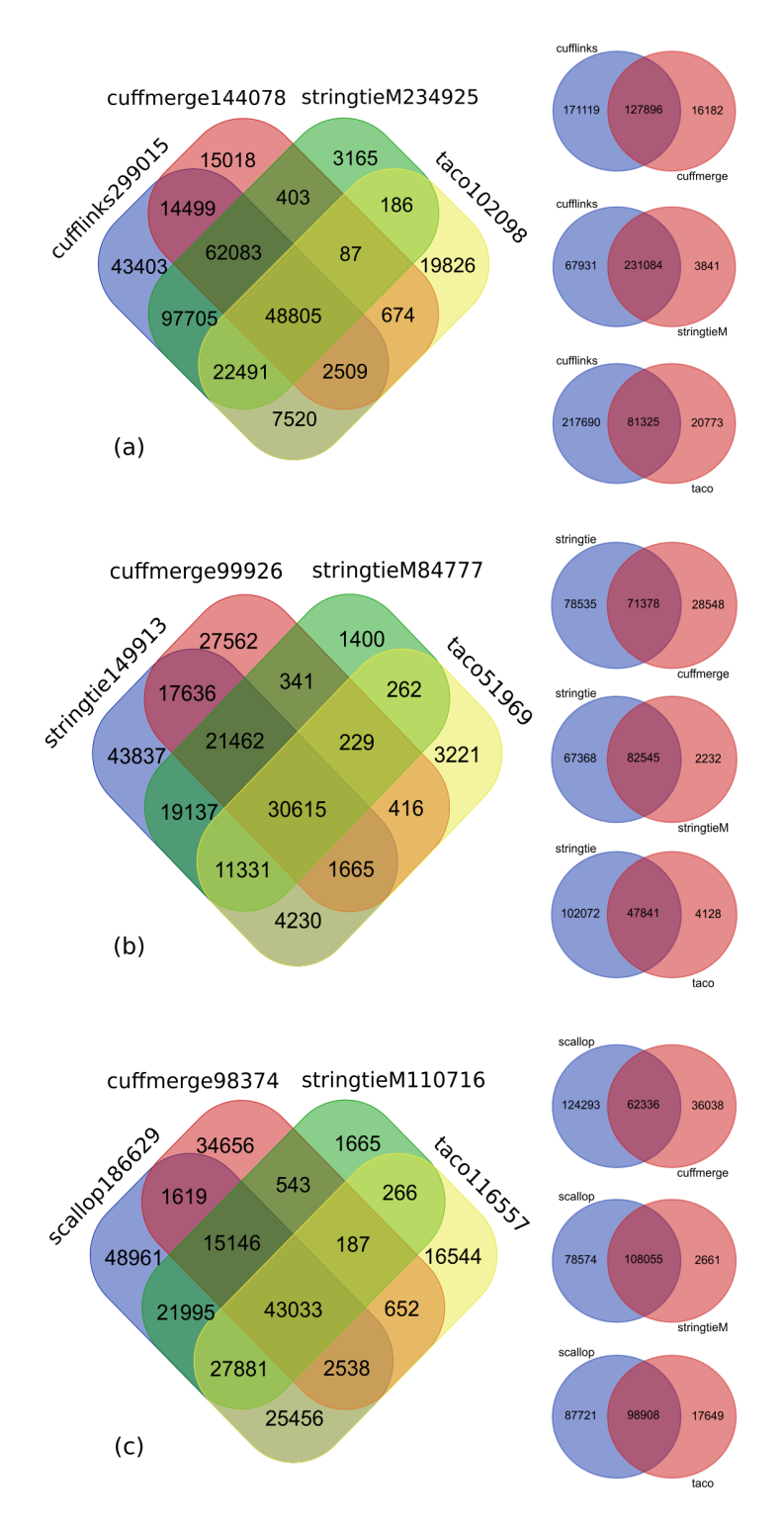


***Figure 9****: Venn diagram of overlapping unique transcripts by intron coordinates of raw assembly of each assembler (a) cufflinks, (b) stringtie and (c) scallop and different merging tools. Venn diagram showing overlapping the number of non-redundant transcripts by intron coordinates of each assembler transcriptome annotation of 18 libraries raw assembly with three merged transcriptome annotation.*


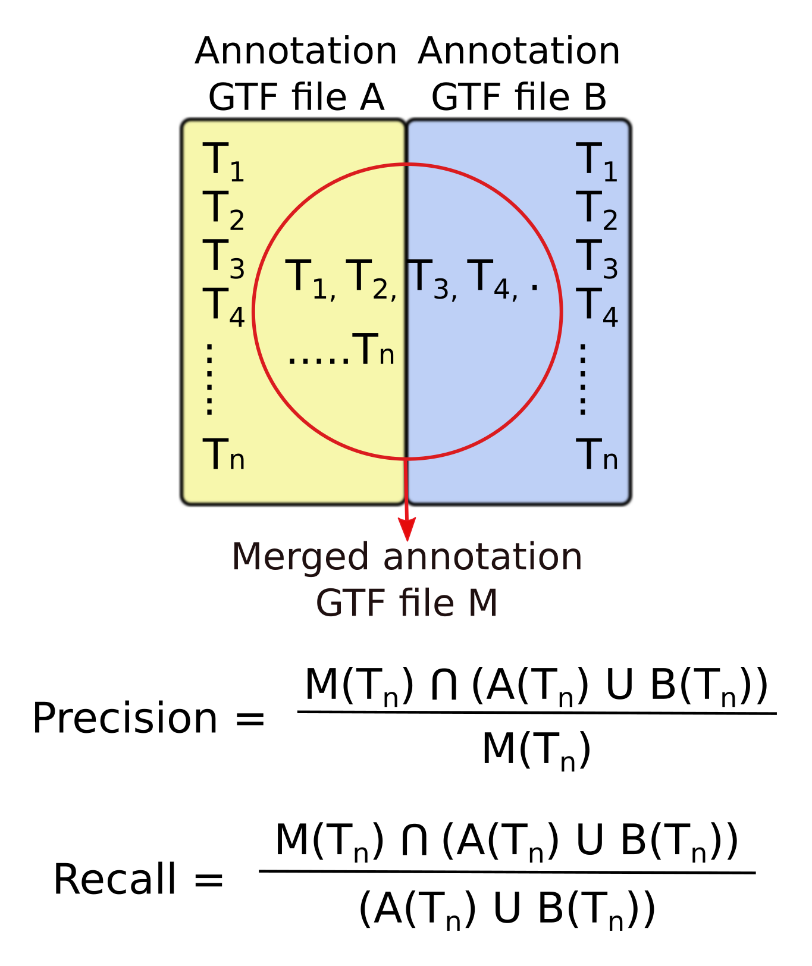


***Figure 10:*** *Venn diagram representing the calculation of precision and recall values of the merged annotation file (M) transcripts with raw assembly annotation files (A and B). The pictorial representation showing calculation formulas of precision and recall values for transcriptome annotation file A, B and its merged transcriptome annotation file M.*

***Figure 11****: The scatter plot showing the calculated precision and recall values of (****a****) unique intronic segments and (****b****) unique transcripts by intronic coordinates between each merged transcriptome annotation and its raw assembly transcriptome annotation. X- and Y- axis represents the precision and recall values respectively.*


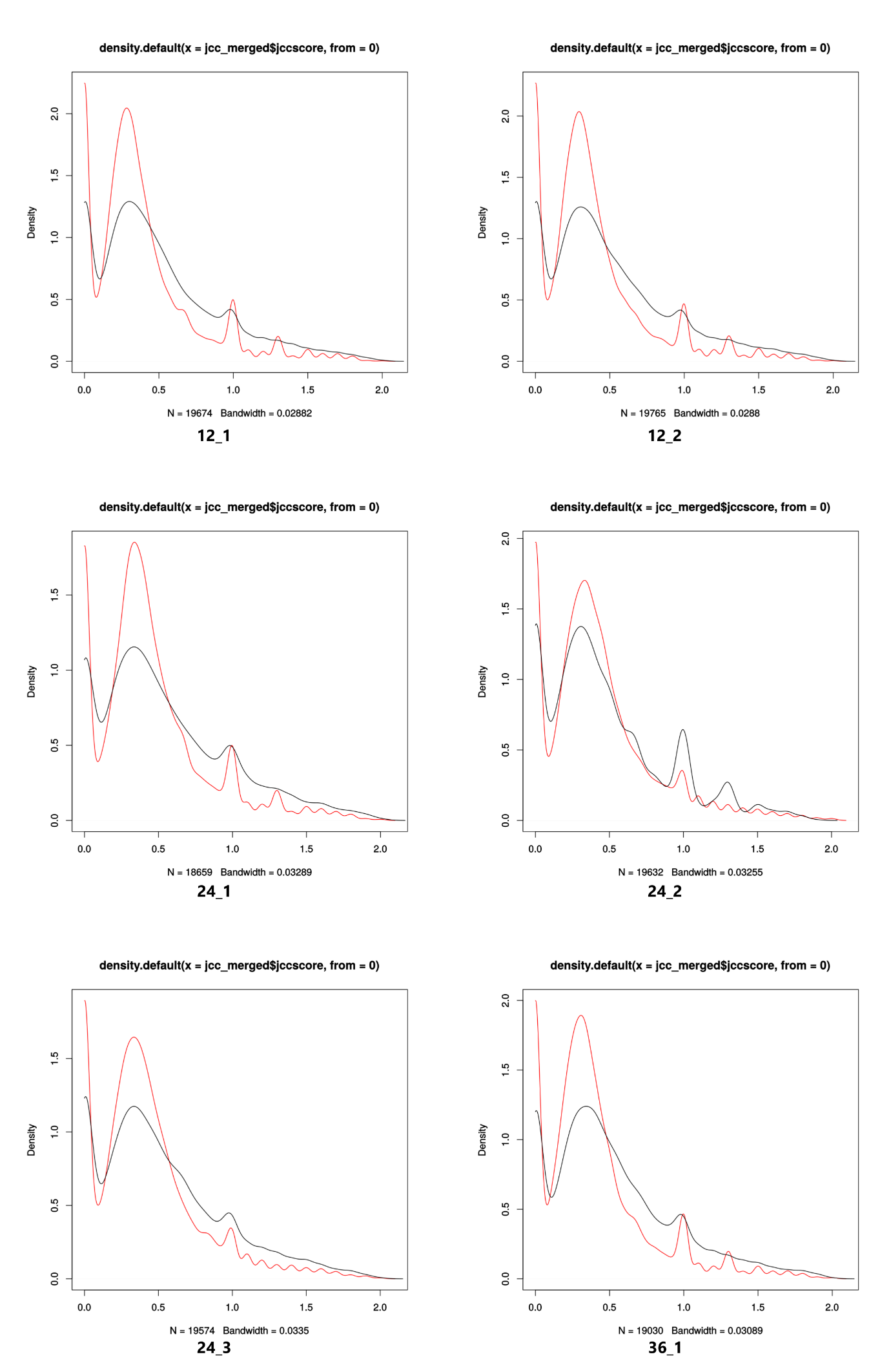


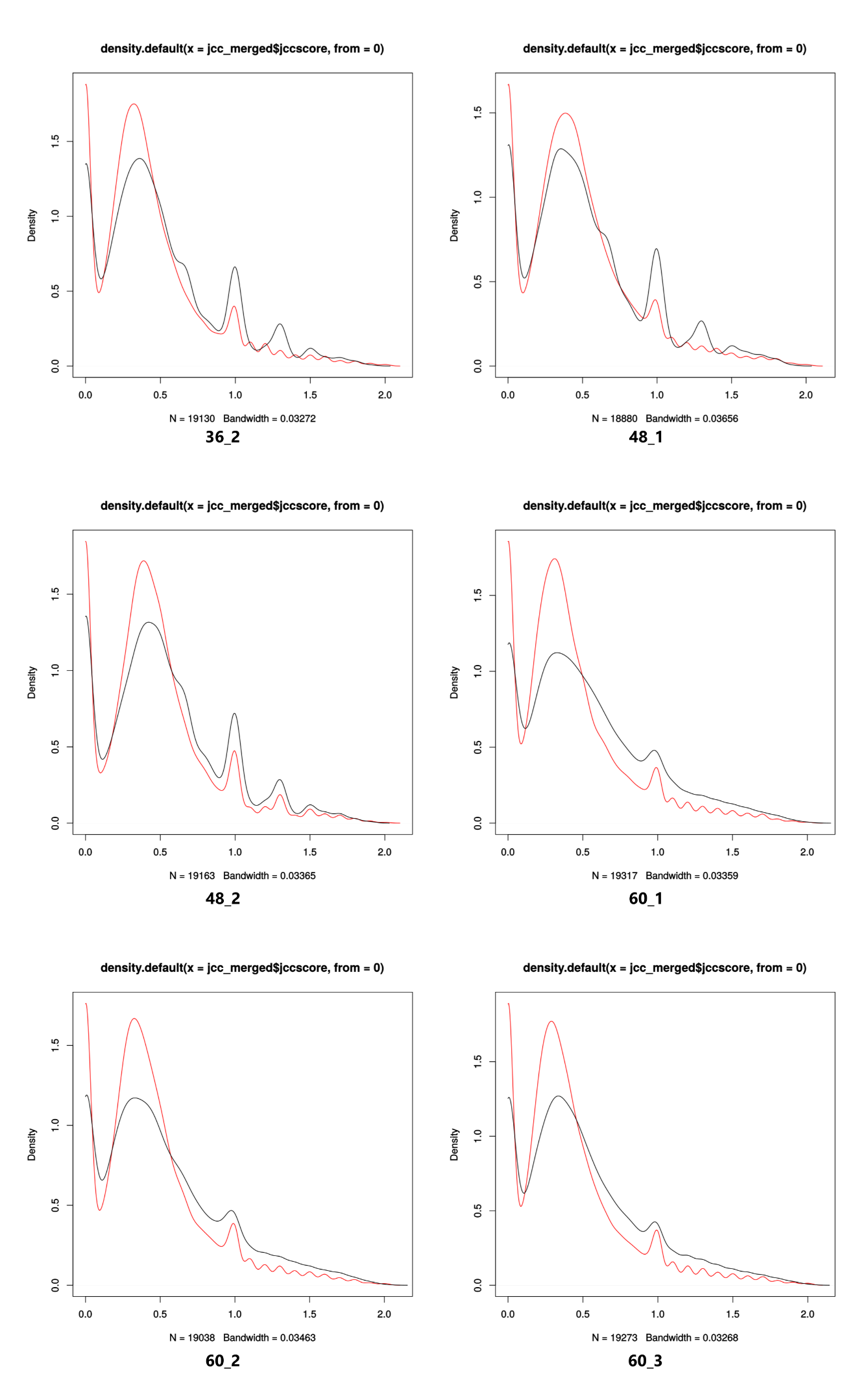


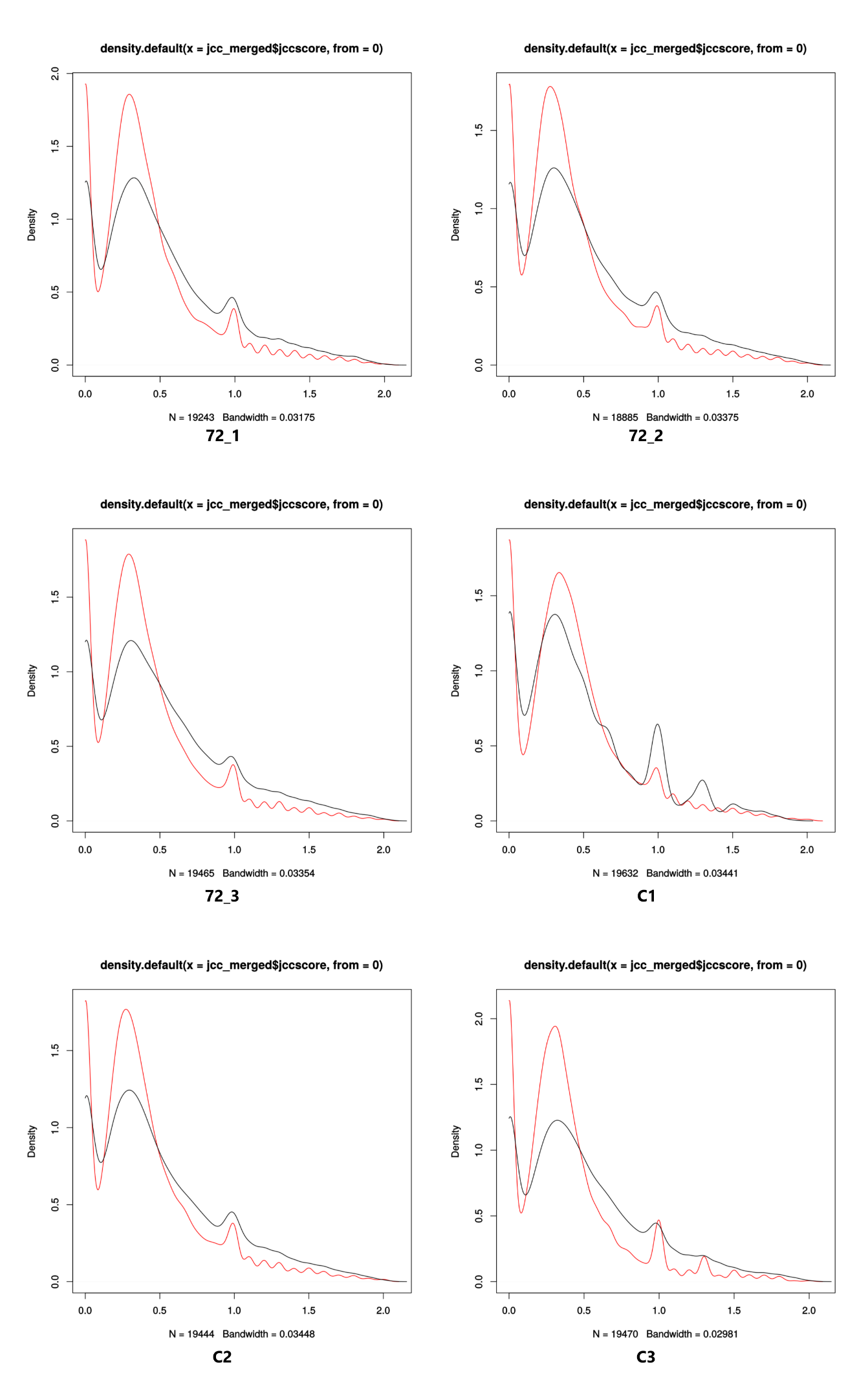


***Figure 12****: Density plots of the JCC scores of Indica RTD (red line) and Ensembl RTD (black line) genes for each sequencing library. The figure showing the comparison of the distribution of genes with JCC scores for both IndicaRTD and Ensembl RTD annotation for each sequencing library. X- and Y- axis represents the JCC score and probability of the genes distributed.*

| **Splice site** | **No. of SJs** |
| --- | --- |
| GT/AG & CT/AC | 296,962 |
| GC/AG & CT/GC | 5,473 |
| AT/AC & GT/AT | 302 |
| Total | 302,737 |

***Table 4:*** *Number of SJs for each canonical splice site. The number of SJs shown for each canonical intron motifs and its equivalents of STAR 2^nd^ pass mapping data.*

| **No. of libraries** | 1 | >=2 | >=3 | >=4 | >=5 | >=6 | >=7 | >=8 | >=9 | >=10 | >=18 | Total |
| --- | --- | --- | --- | --- | --- | --- | --- | --- | --- | --- | --- | --- |
| **No. of SJs** | 5,2152 | 250,585 | 221,829 | 203,206 | 189,634 | 179,164 | 170,445 | 163,100 | 156,365 | 150,437 | 103,710 | 302737 |
| **Percentage** | 17.23 | 82.77 | 73.27 | 67.12 | 62.64 | 59.18 | 56.30 | 53.88 | 51.65 | 49.69 | 34.26 |  |

***Table 5:*** *Number of SJs found in all the libraries. The table showing the number of canonical SJs found in the number of libraries.*

| **Assembler** | **No. of unique total SJs across all the 18 libraries** | **No. of unique SJs matched with 2^nd^ pass mapping unique STAR generated SJs** |
| --- | --- | --- |
| **Cufflinks** | 376,001 | 170,224 |
| **StringTie2** | 180,118 | 178,298 |
| **Scallop** | 169,757 | 167,824 |

***Table 7:*** *Total number of unique and matched SJs of three assemblers data sets. The table showing the number of unique SJs of the cufflinks, stringtie2 & scallop assemblies data sets of the 18 RNA-seq libraries and the number of matching SJs with the STAR 2^nd^ pass mapping generated SJs.*

|  | **cufflinks** | | | **scallop** | | | **stringtie2** | | |
| --- | --- | --- | --- | --- | --- | --- | --- | --- | --- |
|  | **gene** | **transcript** | **exons**  **(unique)** | **gene** | **transcript** | **exons**  **(unique)** | **gene** | **transcript** | **exons**  **(unique)** |
| **cuffmerge** | 54387 | 170241 | 458143 | 47182 | 116172 | 299385 | 49686 | 118487 | 305286 |
| **taco** | 48857 | 126236 | 333329 | 35167 | 132100 | 252749 | 43929 | 70413 | 198608 |
| **stringtieM** | 51012 | 256275 | 750359 | 34455 | 119581 | 302943 | 43564 | 101407 | 278547 |

***Table 8****: Number of genes, transcripts and exons generated. The table showing the total number of genes, transcripts and unique exons of the merged annotations of the three merging tools for the three assemblers annotation files for the 18 RNA-seq libraries individually.*

|  | **cufflinks** | | **scallop** | | **stringtie2** | |
| --- | --- | --- | --- | --- | --- | --- |
|  | No. of transcripts | Percentage | No. of transcripts | Percentage | No. of transcripts | Percentage |
| **cuffmerge** | 25516 | 14.99 | 16390 | 14.11 | 18011 | 15.20 |
| **taco** | 23735 | 18.80 | 12408 | 9.39 | 17945 | 25.49 |
| **stringtieM** | 21350 | 8.33 | 8865 | 7.41 | 16630 | 16.40 |

***Table 9****: Percentage of mono-exonic transcripts. The table showing the number of single exon containing transcripts of the merged assembly annotations of the three merge tools for the three assemblers.*

|  | **cufflinks** | **scallop** | **stringtie2** |  |  |  |  |
| --- | --- | --- | --- | --- | --- | --- | --- |
| **By exon** |  |  |  | **Total** | **1** | **2** | **3** |
| cuffmerge | 170197 | 116153 | 118428 | 315588 | 268542 | 4902 | 42144 |
| taco | 126236 | 132100 | 70413 | 318088 | 307482 | 10551 | 55 |
| stringtieM | 256275 | 119581 | 101407 | 461474 | 445832 | 15495 | 147 |
| **By intron** |  |  |  | **Total** | **1** | **2** | **3** |
| cuffmerge | 144078 | 98374 | 99926 | 230872 | 162561 | 25116 | 43195 |
| taco | 102098 | 116557 | 51969 | 211509 | 170585 | 22733 | 18191 |
| stringtieM | 234925 | 110716 | 84777 | 347014 | 289070 | 32484 | 25460 |

***Table 10****: Number of non-redundant transcripts based on exon and intron co-ordinates*

*column “Total” represents the total number of non-redundant transcripts of three assemblers (cufflinks, scallop & stringtie2); column “1”: transcripts found in any one of the assemblers; column “2”: transcripts found in any two of the assemblers; column “3”: transcripts found in among three assemblers.

| **Intron length** | **1000** | **2000** | **3000** | **4000** | **5000** | **6000** | **7000** | **8000** | **9000** | **10000** | **Total** |
| --- | --- | --- | --- | --- | --- | --- | --- | --- | --- | --- | --- |
| **cuffmerge_cufflinks** | 854451 | 61098 | 12884 | 4867 | 2334 | 1407 | 254 | 230 | 105 | 85 | 937715 |
| **cuffmerge_stringtie** | 571281 | 44411 | 10038 | 3992 | 2130 | 1352 | 167 | 153 | 63 | 53 | 633640 |
| **cuffmerge_scallop** | 521164 | 40382 | 8948 | 3483 | 1819 | 1124 | 178 | 161 | 70 | 56 | 577385 |
| **stringtieM_cufflinks** | 1367972 | 92658 | 19649 | 7041 | 3380 | 2147 | 338 | 327 | 125 | 134 | 1493771 |
| **stringtieM_stringtie** | 415018 | 31202 | 7359 | 3267 | 1855 | 1188 | 83 | 58 | 12 | 24 | 460066 |
| **stringtieM_scallop** | 531292 | 37421 | 7951 | 3314 | 1740 | 1148 | 108 | 66 | 13 | 27 | 583080 |
| **taco_cufflinks** | 534284 | 39481 | 8757 | 3294 | 1579 | 1009 | 189 | 138 | 66 | 58 | 588855 |
| **taco_stringtie** | 230613 | 18088 | 4244 | 1923 | 1108 | 754 | 52 | 34 | 11 | 18 | 256845 |
| **taco_scallop** | 532735 | 34782 | 7374 | 3010 | 1626 | 1060 | 87 | 72 | 13 | 31 | 580790 |

***Table 11****: Length of all introns in merged assemblies. The table showing the number of introns with the lengths containing 1000-10000 range for the merged annotation transcripts generated by three merge tools.*

**
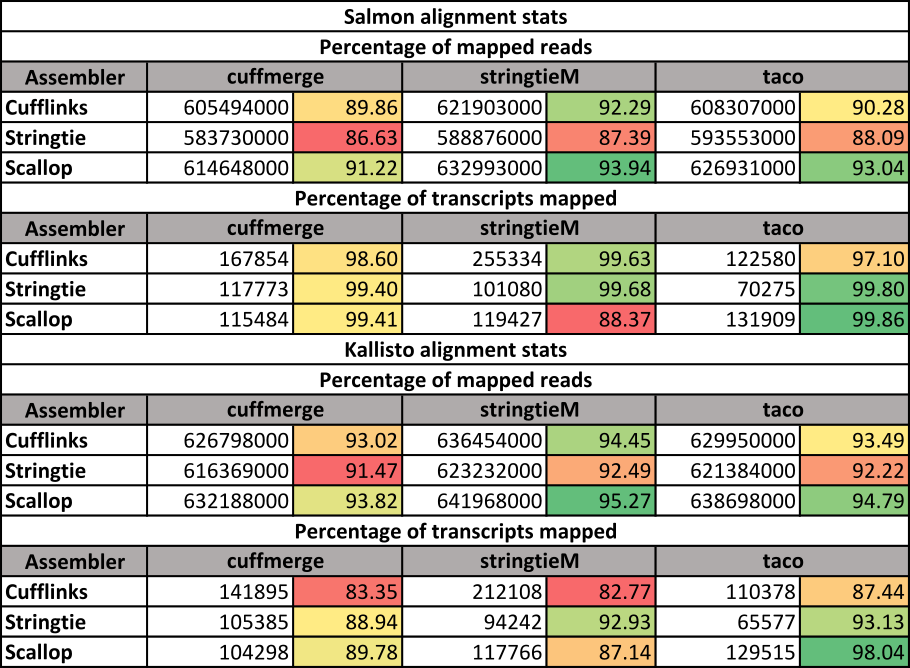
**

***Table 12:*** *Percentage of read mapping rate and transcripts mapped by reads of 9 transcript annotation files using salmon and kallisto. The table showing the percentages of RNA-seq clean reads mapped to the merged annotation transcripts generated by three merge tools for the three different assemblers annotation also showing the percentages of the annotation transcripts mapped by RNA-seq clean reads for both the alignment tools salmon and kallisto.*

| **No. of unique SJ** | | | |
| --- | --- | --- | --- |
|  | **No. of reads** | | |
| **Overhang length** | **>=2** | **>=5** | **>=10** |
| **>=7** | 201,206 | 139,233 | 118,079 |
| **>=10** | 199,637 | 138,532 | 117,540 |
| **>=11** | 199,136 | 138,303 | 117,373 |

***Table 13****: Number of canonical SJs with number of unique mapping reads crossing the junction and overhang length. The table showing the number of canonical SJs with uniquely mapped reads crossing the junction at least 2, 5 & 10 and its overhang length at least 7, 10 & 11.*
