# Supplementary material for: High-quality reference transcriptome construction improves RNA-seq quantification in Oryza sativa indica
Source: Front Genet. 2022 Sep 29;13:995072. doi: 10.3389/fgene.2022.995072 (PMC9558114; doi:10.3389/fgene.2022.995072)
Supplement: Supplementary file 2 [file Presentation1.PPTX]

## Slide 1
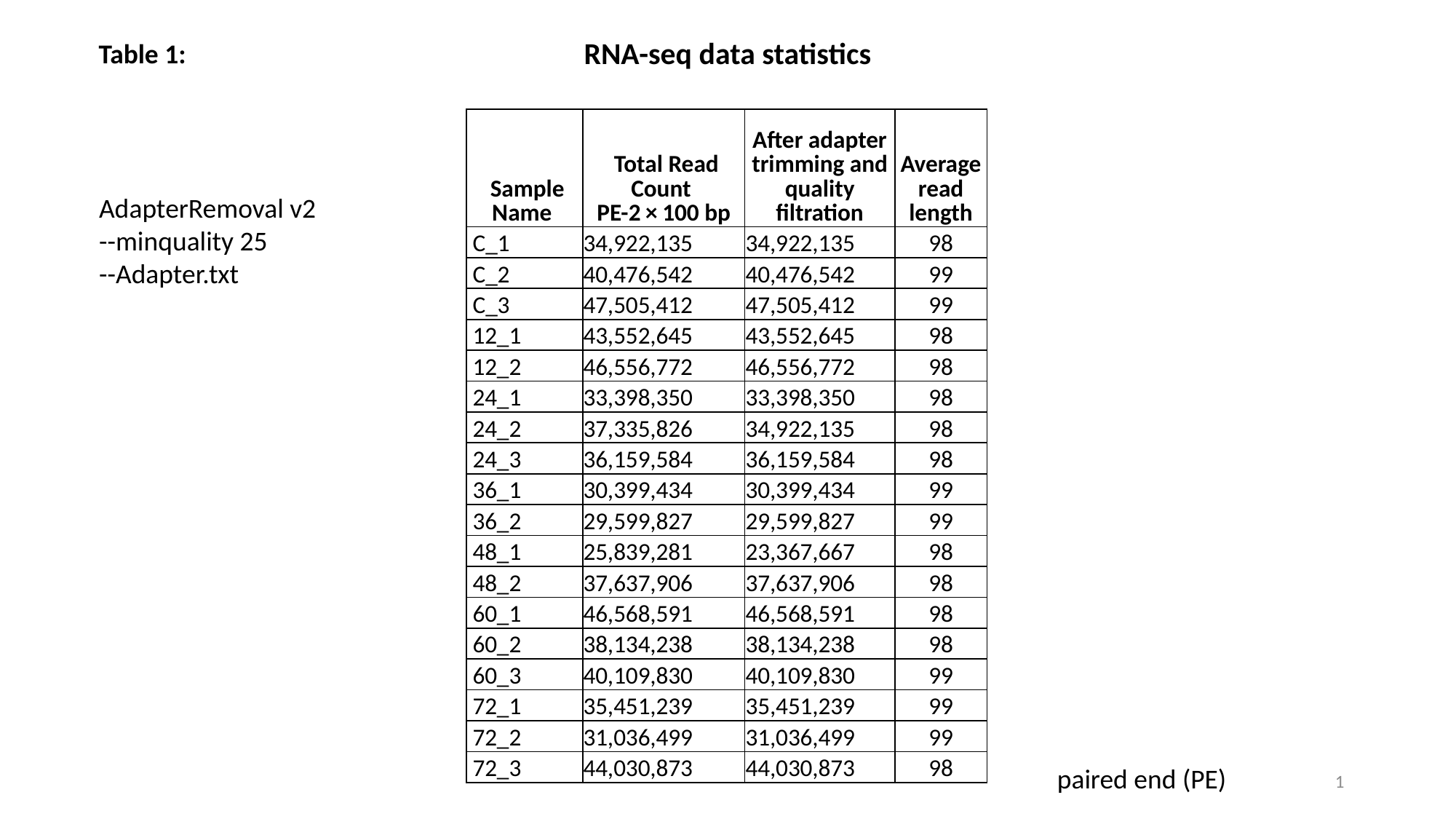

RNA-seq data statistics
Table 1:
| Sample Name | Total Read Count PE-2 × 100 bp | After adapter trimming and quality filtration | Average read length |
| --- | --- | --- | --- |
| C\_1 | 34,922,135 | 34,922,135 | 98 |
| C\_2 | 40,476,542 | 40,476,542 | 99 |
| C\_3 | 47,505,412 | 47,505,412 | 99 |
| 12\_1 | 43,552,645 | 43,552,645 | 98 |
| 12\_2 | 46,556,772 | 46,556,772 | 98 |
| 24\_1 | 33,398,350 | 33,398,350 | 98 |
| 24\_2 | 37,335,826 | 34,922,135 | 98 |
| 24\_3 | 36,159,584 | 36,159,584 | 98 |
| 36\_1 | 30,399,434 | 30,399,434 | 99 |
| 36\_2 | 29,599,827 | 29,599,827 | 99 |
| 48\_1 | 25,839,281 | 23,367,667 | 98 |
| 48\_2 | 37,637,906 | 37,637,906 | 98 |
| 60\_1 | 46,568,591 | 46,568,591 | 98 |
| 60\_2 | 38,134,238 | 38,134,238 | 98 |
| 60\_3 | 40,109,830 | 40,109,830 | 99 |
| 72\_1 | 35,451,239 | 35,451,239 | 99 |
| 72\_2 | 31,036,499 | 31,036,499 | 99 |
| 72\_3 | 44,030,873 | 44,030,873 | 98 |
AdapterRemoval v2
--minquality 25
--Adapter.txt
paired end (PE)
1

## Slide 2
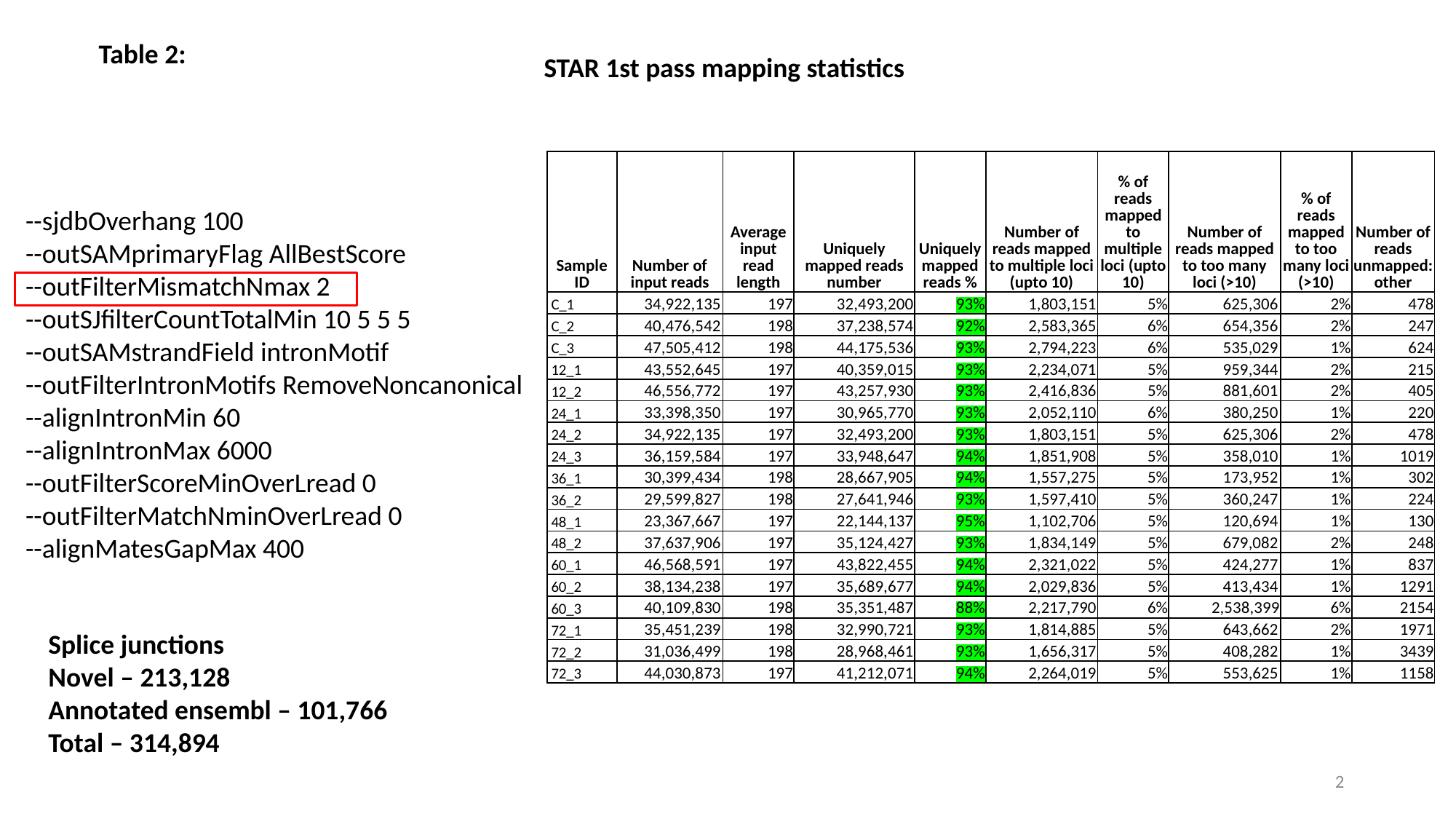

Table 2:
STAR 1st pass mapping statistics
| Sample ID | Number of input reads | Average input read length | Uniquely mapped reads number | Uniquely mapped reads % | Number of reads mapped to multiple loci (upto 10) | % of reads mapped to multiple loci (upto 10) | Number of reads mapped to too many loci (>10) | % of reads mapped to too many loci (>10) | Number of reads unmapped: other |
| --- | --- | --- | --- | --- | --- | --- | --- | --- | --- |
| C\_1 | 34,922,135 | 197 | 32,493,200 | 93% | 1,803,151 | 5% | 625,306 | 2% | 478 |
| C\_2 | 40,476,542 | 198 | 37,238,574 | 92% | 2,583,365 | 6% | 654,356 | 2% | 247 |
| C\_3 | 47,505,412 | 198 | 44,175,536 | 93% | 2,794,223 | 6% | 535,029 | 1% | 624 |
| 12\_1 | 43,552,645 | 197 | 40,359,015 | 93% | 2,234,071 | 5% | 959,344 | 2% | 215 |
| 12\_2 | 46,556,772 | 197 | 43,257,930 | 93% | 2,416,836 | 5% | 881,601 | 2% | 405 |
| 24\_1 | 33,398,350 | 197 | 30,965,770 | 93% | 2,052,110 | 6% | 380,250 | 1% | 220 |
| 24\_2 | 34,922,135 | 197 | 32,493,200 | 93% | 1,803,151 | 5% | 625,306 | 2% | 478 |
| 24\_3 | 36,159,584 | 197 | 33,948,647 | 94% | 1,851,908 | 5% | 358,010 | 1% | 1019 |
| 36\_1 | 30,399,434 | 198 | 28,667,905 | 94% | 1,557,275 | 5% | 173,952 | 1% | 302 |
| 36\_2 | 29,599,827 | 198 | 27,641,946 | 93% | 1,597,410 | 5% | 360,247 | 1% | 224 |
| 48\_1 | 23,367,667 | 197 | 22,144,137 | 95% | 1,102,706 | 5% | 120,694 | 1% | 130 |
| 48\_2 | 37,637,906 | 197 | 35,124,427 | 93% | 1,834,149 | 5% | 679,082 | 2% | 248 |
| 60\_1 | 46,568,591 | 197 | 43,822,455 | 94% | 2,321,022 | 5% | 424,277 | 1% | 837 |
| 60\_2 | 38,134,238 | 197 | 35,689,677 | 94% | 2,029,836 | 5% | 413,434 | 1% | 1291 |
| 60\_3 | 40,109,830 | 198 | 35,351,487 | 88% | 2,217,790 | 6% | 2,538,399 | 6% | 2154 |
| 72\_1 | 35,451,239 | 198 | 32,990,721 | 93% | 1,814,885 | 5% | 643,662 | 2% | 1971 |
| 72\_2 | 31,036,499 | 198 | 28,968,461 | 93% | 1,656,317 | 5% | 408,282 | 1% | 3439 |
| 72\_3 | 44,030,873 | 197 | 41,212,071 | 94% | 2,264,019 | 5% | 553,625 | 1% | 1158 |
--sjdbOverhang 100
--outSAMprimaryFlag AllBestScore
--outFilterMismatchNmax 2
--outSJfilterCountTotalMin 10 5 5 5
--outSAMstrandField intronMotif
--outFilterIntronMotifs RemoveNoncanonical
--alignIntronMin 60
--alignIntronMax 6000
--outFilterScoreMinOverLread 0
--outFilterMatchNminOverLread 0
--alignMatesGapMax 400
Splice junctions
Novel – 213,128
Annotated ensembl – 101,766
Total – 314,894‬
2

## Slide 3
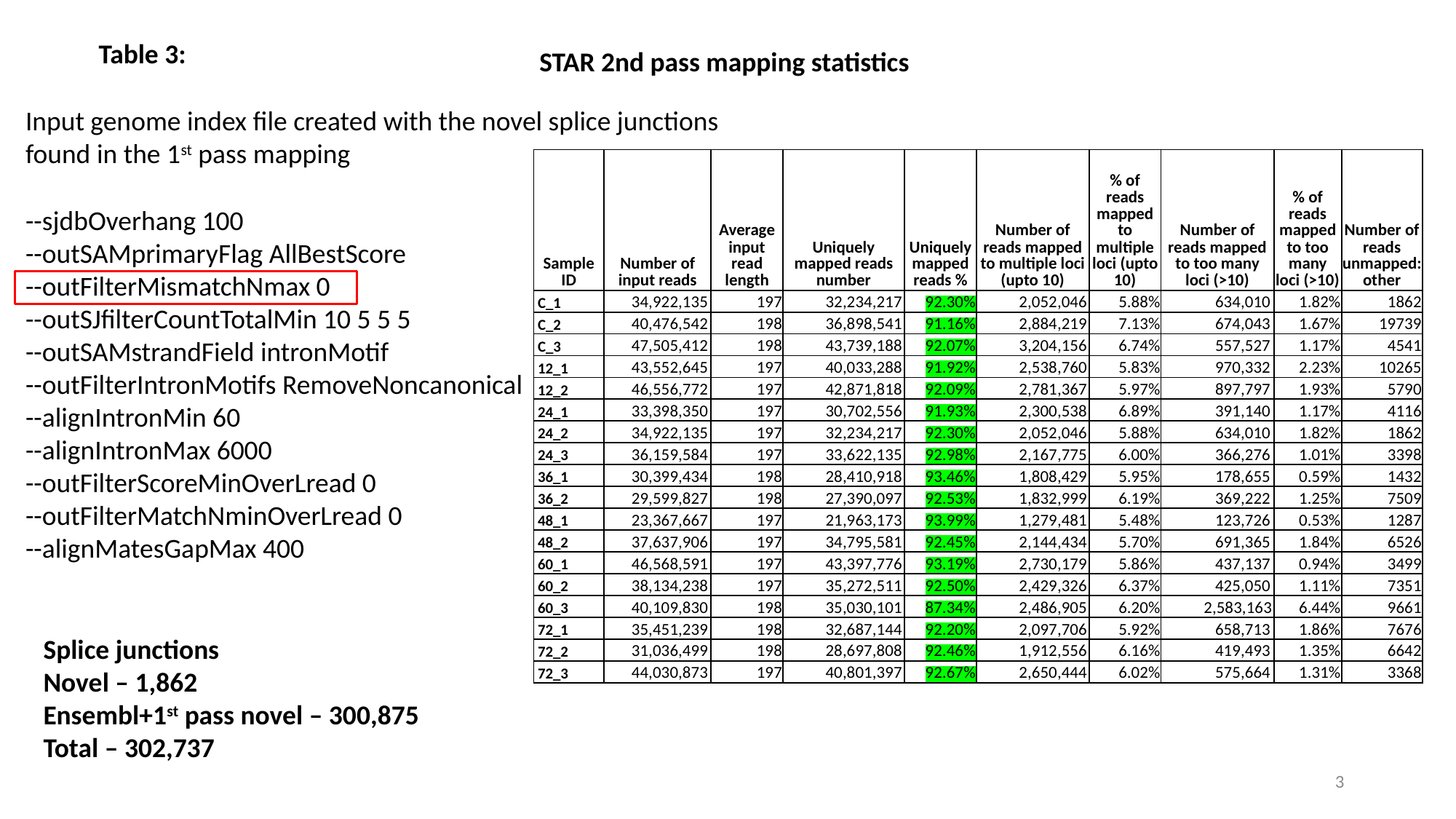

Table 3:
STAR 2nd pass mapping statistics
Input genome index file created with the novel splice junctions found in the 1st pass mapping
| Sample ID | Number of input reads | Average input read length | Uniquely mapped reads number | Uniquely mapped reads % | Number of reads mapped to multiple loci (upto 10) | % of reads mapped to multiple loci (upto 10) | Number of reads mapped to too many loci (>10) | % of reads mapped to too many loci (>10) | Number of reads unmapped: other |
| --- | --- | --- | --- | --- | --- | --- | --- | --- | --- |
| C\_1 | 34,922,135 | 197 | 32,234,217 | 92.30% | 2,052,046 | 5.88% | 634,010 | 1.82% | 1862 |
| C\_2 | 40,476,542 | 198 | 36,898,541 | 91.16% | 2,884,219 | 7.13% | 674,043 | 1.67% | 19739 |
| C\_3 | 47,505,412 | 198 | 43,739,188 | 92.07% | 3,204,156 | 6.74% | 557,527 | 1.17% | 4541 |
| 12\_1 | 43,552,645 | 197 | 40,033,288 | 91.92% | 2,538,760 | 5.83% | 970,332 | 2.23% | 10265 |
| 12\_2 | 46,556,772 | 197 | 42,871,818 | 92.09% | 2,781,367 | 5.97% | 897,797 | 1.93% | 5790 |
| 24\_1 | 33,398,350 | 197 | 30,702,556 | 91.93% | 2,300,538 | 6.89% | 391,140 | 1.17% | 4116 |
| 24\_2 | 34,922,135 | 197 | 32,234,217 | 92.30% | 2,052,046 | 5.88% | 634,010 | 1.82% | 1862 |
| 24\_3 | 36,159,584 | 197 | 33,622,135 | 92.98% | 2,167,775 | 6.00% | 366,276 | 1.01% | 3398 |
| 36\_1 | 30,399,434 | 198 | 28,410,918 | 93.46% | 1,808,429 | 5.95% | 178,655 | 0.59% | 1432 |
| 36\_2 | 29,599,827 | 198 | 27,390,097 | 92.53% | 1,832,999 | 6.19% | 369,222 | 1.25% | 7509 |
| 48\_1 | 23,367,667 | 197 | 21,963,173 | 93.99% | 1,279,481 | 5.48% | 123,726 | 0.53% | 1287 |
| 48\_2 | 37,637,906 | 197 | 34,795,581 | 92.45% | 2,144,434 | 5.70% | 691,365 | 1.84% | 6526 |
| 60\_1 | 46,568,591 | 197 | 43,397,776 | 93.19% | 2,730,179 | 5.86% | 437,137 | 0.94% | 3499 |
| 60\_2 | 38,134,238 | 197 | 35,272,511 | 92.50% | 2,429,326 | 6.37% | 425,050 | 1.11% | 7351 |
| 60\_3 | 40,109,830 | 198 | 35,030,101 | 87.34% | 2,486,905 | 6.20% | 2,583,163 | 6.44% | 9661 |
| 72\_1 | 35,451,239 | 198 | 32,687,144 | 92.20% | 2,097,706 | 5.92% | 658,713 | 1.86% | 7676 |
| 72\_2 | 31,036,499 | 198 | 28,697,808 | 92.46% | 1,912,556 | 6.16% | 419,493 | 1.35% | 6642 |
| 72\_3 | 44,030,873 | 197 | 40,801,397 | 92.67% | 2,650,444 | 6.02% | 575,664 | 1.31% | 3368 |
--sjdbOverhang 100
--outSAMprimaryFlag AllBestScore
--outFilterMismatchNmax 0
--outSJfilterCountTotalMin 10 5 5 5
--outSAMstrandField intronMotif
--outFilterIntronMotifs RemoveNoncanonical
--alignIntronMin 60
--alignIntronMax 6000
--outFilterScoreMinOverLread 0
--outFilterMatchNminOverLread 0
--alignMatesGapMax 400
Splice junctions
Novel – 1,862
Ensembl+1st pass novel – 300,875
Total – 302,737
3

## Slide 4
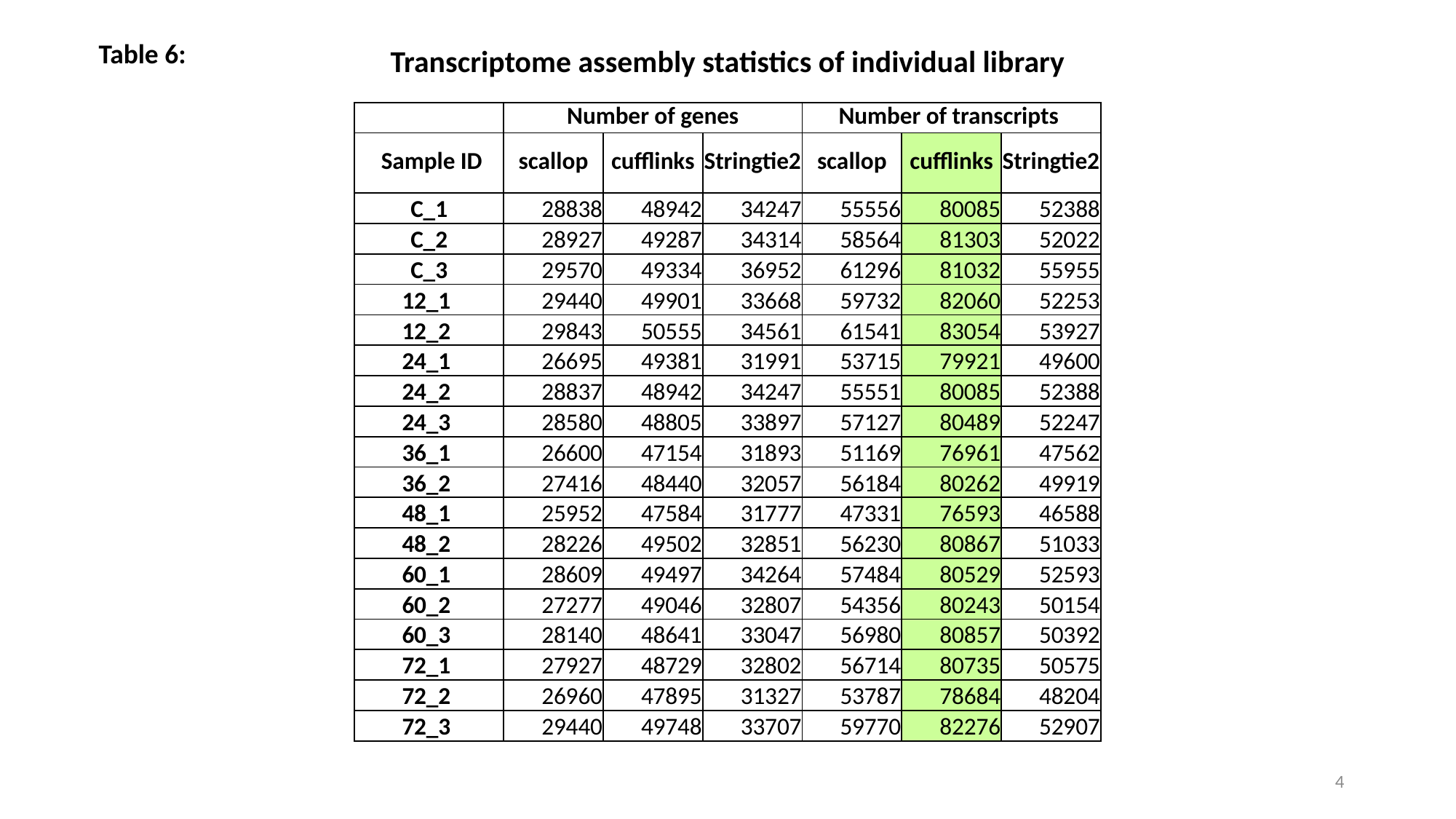

Table 6:
Transcriptome assembly statistics of individual library
| | Number of genes | | | Number of transcripts | | |
| --- | --- | --- | --- | --- | --- | --- |
| Sample ID | scallop | cufflinks | Stringtie2 | scallop | cufflinks | Stringtie2 |
| C\_1 | 28838 | 48942 | 34247 | 55556 | 80085 | 52388 |
| C\_2 | 28927 | 49287 | 34314 | 58564 | 81303 | 52022 |
| C\_3 | 29570 | 49334 | 36952 | 61296 | 81032 | 55955 |
| 12\_1 | 29440 | 49901 | 33668 | 59732 | 82060 | 52253 |
| 12\_2 | 29843 | 50555 | 34561 | 61541 | 83054 | 53927 |
| 24\_1 | 26695 | 49381 | 31991 | 53715 | 79921 | 49600 |
| 24\_2 | 28837 | 48942 | 34247 | 55551 | 80085 | 52388 |
| 24\_3 | 28580 | 48805 | 33897 | 57127 | 80489 | 52247 |
| 36\_1 | 26600 | 47154 | 31893 | 51169 | 76961 | 47562 |
| 36\_2 | 27416 | 48440 | 32057 | 56184 | 80262 | 49919 |
| 48\_1 | 25952 | 47584 | 31777 | 47331 | 76593 | 46588 |
| 48\_2 | 28226 | 49502 | 32851 | 56230 | 80867 | 51033 |
| 60\_1 | 28609 | 49497 | 34264 | 57484 | 80529 | 52593 |
| 60\_2 | 27277 | 49046 | 32807 | 54356 | 80243 | 50154 |
| 60\_3 | 28140 | 48641 | 33047 | 56980 | 80857 | 50392 |
| 72\_1 | 27927 | 48729 | 32802 | 56714 | 80735 | 50575 |
| 72\_2 | 26960 | 47895 | 31327 | 53787 | 78684 | 48204 |
| 72\_3 | 29440 | 49748 | 33707 | 59770 | 82276 | 52907 |
4
